# Histone Methylation Related Therapeutic Challenge in Cardiovascular Diseases

**DOI:** 10.3389/fcvm.2021.710053

**Published:** 2021-09-09

**Authors:** Yang Yang, Ying Luan, Rui-Xia Yuan, Yi Luan

**Affiliations:** ^1^Department of Translational Medicine Center, The First Affiliated Hospital of Zhengzhou University, Zhengzhou, China; ^2^Department of Physiology and Neurobiology, School of Basic Medical Sciences, Zhengzhou University, Zhengzhou, China

**Keywords:** cardiovascular diseases, demethylation, methyltransferases, demethylases, histone-methylation

## Abstract

The epidemic of cardiovascular diseases (CVDs) is predicted to spread rapidly in advanced countries accompanied by the high prevalence of risk factors. In terms of pathogenesis, the pathophysiology of CVDs is featured by multiple disorders, including vascular inflammation accompanied by simultaneously perturbed pathways, such as cell death and acute/chronic inflammatory reactions. Epigenetic alteration is involved in the regulation of genome stabilization and cellular homeostasis. The association between CVD progression and histone modifications is widely known. Among the histone modifications, histone methylation is a reversible process involved in the development and homeostasis of the cardiovascular system. Abnormal methylation can promote CVD progression. This review discusses histone methylation and the enzymes involved in the cardiovascular system and determine the effects of histone methyltransferases and demethylases on the pathogenesis of CVDs. We will further demonstrate key proteins mediated by histone methylation in blood vessels and review histone methylation-mediated cardiomyocytes and cellular functions and pathways in CVDs. Finally, we will summarize the role of inhibitors of histone methylation and demethylation in CVDs and analyze their therapeutic potential, based on previous studies.

## Introduction

As a major trigger of mortality worldwide, the epidemic of cardiovascular diseases (CVDs) is predicted to spread rapidly in developing and developed countries along with the high prevalence of risk factors, including hypertension, diabetes, and obesity (1). In 2016, CVDs caused ~17.9 million deaths globally (2). The mortality of CVDs worldwide is estimated to reach nearly 23.6 million in 2030 (3). Several risk factors, both genetic and behavioral, including diabetes, high blood pressure, high cholesterol, smoking, unhealthy nutrition, obesity, physical inactivity, aging, and arterial hypertension, account for the occurrence of CVDs (4). The clinical features of CVDs mainly include vascular inflammation, endothelial dysfunction, atherosclerosis, fibrosis, and thrombosis accompanied by multiple simultaneously perturbed pathways, such as cell death and acute/chronic inflammatory reactions (5).

The structural and functional abnormalities of the heart and blood vessels mainly cause CVDs. The heart is composed of several types of cells, mainly including cardiomyocytes and fibroblasts, and an intricate network of blood vessels made up of fibroblasts, connective tissues, smooth muscle cells, and endothelial cells [ECs; (6)]. Considering the complex composition, the dysfunction of these cells in the heart and vasculature contributes to the pathogenesis of CVDs. CVDs might be triggered by multiple processes, such as mitochondrial dysfunction, reactive oxygen species formation, abnormal calcium homeostasis, deleterious phosphorylation signaling, proteostasis imbalance, dysregulated nutrient sensing, cellular senescence, stem cell exhaustion, genomic instability, telomere attrition, and epigenetic alterations (7, 8). With the rapid advance in biochemical, molecular, and high-throughput sequencing technologies, the dysregulated expression profiles of the human genome in CVD patients have focused on (9). However, dynamic alterations in the gene expression landscape can contribute to the progression of CVDs (10). The dynamic gene expression landscape is subject to different levels of regulation, including genetics, epitranscriptomics, transcriptomics, and epigenetics (11). Epigenetics provides the link between genetic programming and environmental influence that results in the expressed phenotype (12). Epigenetics plays a major role in the occurrence and progression of several CVDs, such as cardiac hypertrophy, heart failure, ischemic heart disease, aortic aneurysm, vascular calcification, and pulmonary hypertension, by mediating gene expression and cellular function (13). Furthermore, epigenetics implies the heritable alteration in the gene expression landscape without alterations in DNA sequence caused by the changes in nucleosome remodeling, which represents the architecture of chromatin and regulates the accessibility of DNA (14). The altered nucleosome remodeling is attributed to the interaction between the environment and the genome (15).

Preliminary studies have pointed to the complex association between CVDs and epigenetic modifications, including DNA methylation, histone modifications, and RNA-based mechanisms (16). Histone modification is the methylation, acetylation, ubiquitination, phosphorylation, SUMOylation, GlcNAcylation, carbonylation, and ADP-ribosylation of histones, H2A, H2B, H3, and H4 (17). Post-translational modifications (PTMs) in core histones effectively modulate the activation and inhibition state of downstream gene transcription (18, 19). For example, H3K4 methylation can activate the expression of α-MHC gene in the left ventricle (LV) compared with that in the right ventricle (RV) (20). Generally, PTMs can be added and removed by specific enzymes, including “writers,” which add modifiers, and “erasers,” which remove modifiers (21). Histone acetylation is added to lysine residues by histone acetyltransferases (HATs) and removed by histone deacetylases [HDACs; (22)]. The aberrant regulation of epigenetic regulators in PTMs is a predisposing factor for cardiac diseases (23). Considering the close involvement of epigenetics in the expression of genes associated with CVDs, the epigenetic mechanism and its critical role in modulating CVD progression should be determined (24). A better understanding of the modulatory mechanism in CVD development may contribute to the discovery of novel therapeutic targets to provide beneficial effects for patients. Pharmacologically targeting epigenetic modification for the treatment of CVDs has been developed and successfully tested in preclinical models.

## Histone Methylation Modification

Alterations at the epigenetic level that mediate chromatin structure are involved in the regulation of genome stabilization and cellular homeostasis (25, 26). In eukaryotic nuclei, DNA is wrapped around by four core histone proteins, namely, H2A, H2B, H3, and H4, which further forms nucleosomes and chromatin (27, 28). Histone modifications alter the structure of nucleosomes, regulate gene transcription, and mediate growth and disease pathogenesis (29, 30). The important and unique roles of these histone modifications have been reported by a number of studies (31, 32).

**Figure 1:**
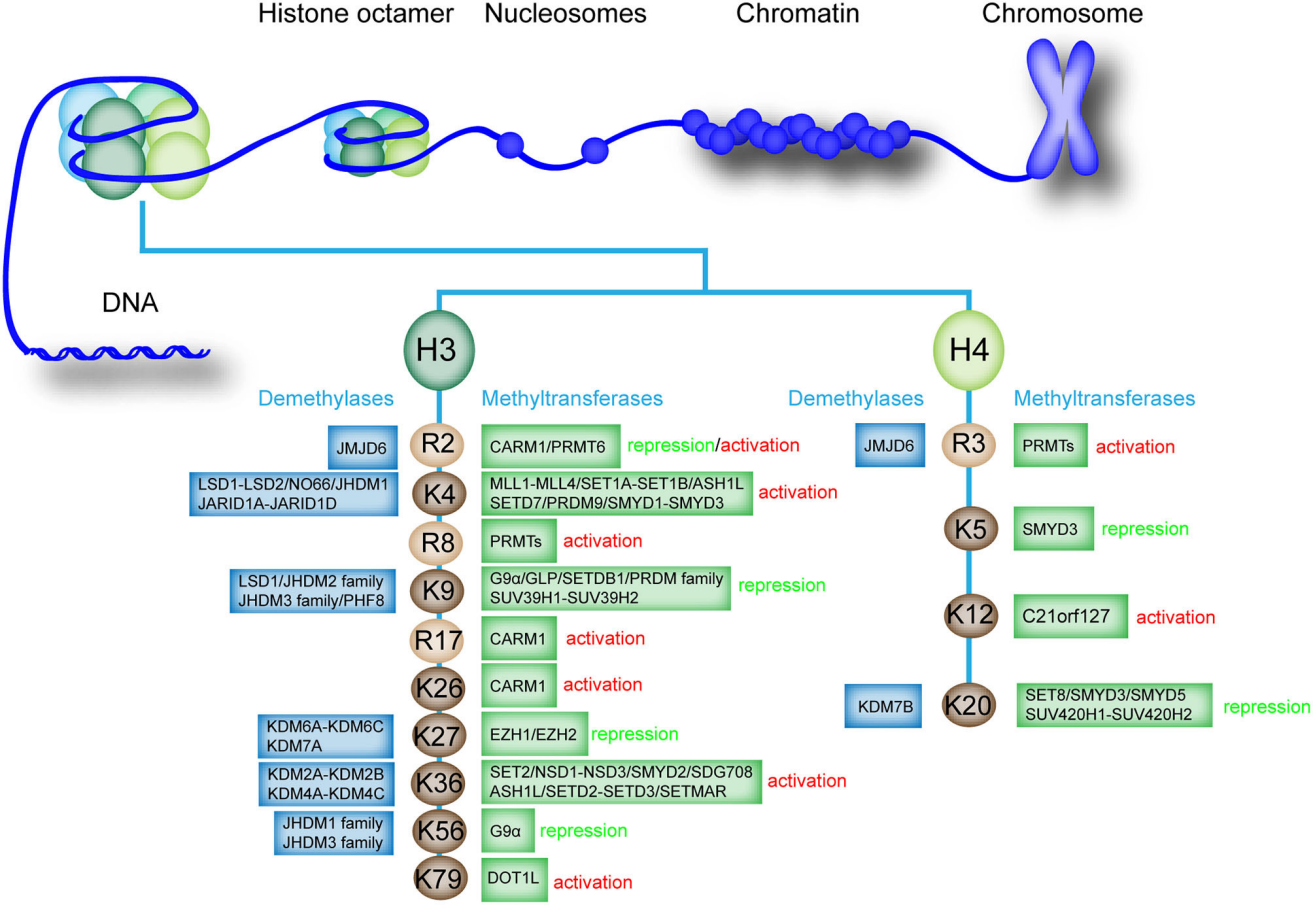
Existing methylation sites in histones, the enzymes involved, and the effects on the transcription of downstream genes. The existing methylation sites and the histone methyltransferases and demethylases that catalyze monomethylation, dimethylation, trimethylation, or demethylation are listed. The effect of methylation modification at each site on downstream gene transcription is labeled as “activation” or “repression”.

Histone methylation is an essential modification that can cause monomethylation (me1), dimethylation (me2), and trimethylation (me3) of several amino acids, thus directly affecting heterochromatin formation, gene imprinting, X chromosome inactivation, and gene transcriptional regulation (33). In general, lysine (Lys or K), arginine (Arg or R), and rarely histidine (His or H) are the most common histone methyl acceptors (30, 34, 35). Histone methylation only occurs at specific lysine and arginine sites of histone H3 and H4 (36). In histone H3, lysine 4, 9, 26, 27, 36, 56, and 79 and arginine 2, 8, and 17 can be methylated. By comparison, histone H4 has fewer methylation sites, in which only lysine 5, 12, and 20 and arginine 3 can be methylated (37, 38). However, the methylation of H2A and H2B in histone octamer has not been confirmed (Figure 1). Histone methylation can occur at distinct positions with divergent transcriptional activity (39). Histone methylation is often associated with transcriptional activation or inhibition of downstream genes (40, 41). The methylation of histone H3K4, R8, R17, K26, K36, K79, H4R3, and K12 can activate gene transcription (42, 43). However, the methylation of histone H3K9, K27, K56, H4K5, and K20 inhibits gene transcription, confirming the complexity of epigenetic regulation of histone methylation (44). Interestingly, under different conditions, the methylation of histone H3R2 can activate and inhibit transcription (33).

Histone methylation is a reversible process that promotes homeostasis in healthy organisms (36). Histone methyltransferases and histone demethylases promote monomethylation, dimethylation, trimethylation, or demethylation of histones (38, 45). Histone methyltransferases, particularly histone lysine methyltransferases (KMTs), are involved in the transfer of methyl group from S-adenosylmethionine to N-terminal tails of lysine residues present on histone (46). Histone demethylases such as lysine-specific demethylase 1 (LSD1) can regulate histone demethylation (47). Histone H3 and H4 can undergo methylation modification, and the methylation and demethylation of different sites are mediated by specific enzymes (Figure 1).

In humans, the following two protein domains carry out lysine methylation: SET domain [named after three *Drosophila melanogaster* proteins originally recognized as containing this domain, namely, Su(var)3–9, Enhancer of zeste, and Trithorax] and the seven beta-strand (7βS) domain [non-SET-domain enzymes; (30, 31)]. These two families account for more than 200 enzymes with different amino acid residue specificity (48). Histone demethylases (HDMs) also include two groups in eukaryotes, including the LSD1 family and the Jumonji C-domain-containing family [JHDMs; (49)]. LSD1 is the first identified histone demethylase (50). HDMs in JHDMs include Fe^2+^- and α-ketoglutarate-dependent hydroxylases, and seven phylogenetically distinct subfamilies were identified in this family (51).

In human cells, the methylation and demethylation of different histone sites are mediated by different enzymes, which precisely regulate histone methylation and gene expression (52). For example, various histone methyltransferases regulate the methylation of histone H3K4, such as mixed lineage leukemia protein 1 (MLL1)-MLL4, SET domain containing 1A (SET1A)-SET1B, and SET domain and MYND domain protein 1 (SMYD1)-SMYD3 (27, 36, 53, 54). Several histone demethylases mediate the demethylation of H3K4, such as proteins in the LSD family and Jumonji, and AT rich interactive domain 1D (JARID) family (55). We further summarized the methyltransferases and demethylases involved in the histone methylation regulation of different sites (Figure 1). Notably, the specific histone demethylase that regulates the demethylation of histone H3R8, R17, K26, K79, H4K5, and K12 has not been determined.

The crosstalk between miRNAs and histone modification forms closed epigenetic machinery loops. Histone modification may activate or inhibit miRNA expression. HDAC inhibition upregulates miR-124 accompanied by the inhibition of the expression of downstream targets, such as CDK4, CDK6, and EZH2 (56). miRNA may also regulate histone modifications. HDAC1 is regulated by miR-34a via binding to the 3′-UTR of HDAC1 mRNA in the foam cells. The overexpression of miR-34a represses the expression of HDAC1 and increases the acetylation levels of H3K9ac, causing aberrant lipid accumulation in the foam cell (57).

## Histone Methylation In CVD Progression

Generally, histones are featured by their large quantity and various modification residues (46, 58). At least eight modifications have been identified in histones, and these modifications are catalyzed by distinct enzymes (59, 60). A genome-wide analysis depicted that 596 out of 1,109 differentially regulated genes harbor at least one histone modifier at the promoter region in adult mouse cardiomyocytes under hypertrophic remodeling, suggesting a key function in the epigenetic landscape in the transcriptome reprogramming of hypertrophic cardiomyocytes (27, 61). Histone modifications (e.g., methylation or acetylation) affect the progression of various forms of CVDs (22). The function of histone modification on target gene modulation specifically relies on cell types and epigenetic marks (62). Epigenetic modifications widely affect CVDs, and the epigenetic modifications involved in CVD progression are listed in Table 1.

**Table 1:** The known methyltransferases and demethylases involved in CVDs progression.

**Regulators**	**Actions**	**Effect**
SMYD1/2/3	Methylation of H3K4, H3K36	SMYD1: Mice: Disrupted right ventricle formation and cardiomyocyte maturation; SMYD3: Zebrafish: Abnormal looping of heart tube, pericardial edema
COMPASS (Ash2, WDR5)	Methylation of H3K4	Involved in vasoconstriction, endothelial dysfunction, and development in numerous cardiovascular diseases
SETD7	Methylation of H3K4	Zebrafish: Developmental heart edema
MLL3	Methylation of H3K4me2	Patients with dilated cardiomyopathy
MLL2	Methylation of H3K4	Zebrafish: Abnormal development of the atria and/or ventricle, prominent bulging of the myocardial wall Mouse: Embryonic lethal, disorganized interventricular septum Human: Kabuki syndrome, congenital heart defects
G9a/EHMT2	Methylation of H3K9me2 and H3K27me3 (lesser extent)	Maintain cardiomyocyte homeostasis and interact with MEF2C to silence the fetal gene program in the adult heart Promote cardiac hypertrophy in stressed hearts
Blimp-1/PRDM	Methylation of H3K9	Mice: Ventricular septal defect and persistent arterial trunk
EHMT1/2	Methylation of H3K9	Protects mice from LVH induced by pressure overload
PRMT5	Methylation of H3R2, H2AR3, and H4R3	Regulate hypertrophic growth via GATA4
EZH2	Methylation of H3K27	Mouse: Failure of myocardial compaction, hypertrabeculation, and ventricular and atrial septal defects
NSD1	Methylation of H3K36	Sotos syndrome
DOT1L	Methylation of H3K79me	Reduction of DOT1L activity causes DCM
PTIP	Co-factor of H3K4 methylation Regulates the expression of Kcnip2	Misregulation of PTIP cause cardiac hypertrophy and failure
LSD1	Demethylation of H3K4	Mice: Ventricular septal defects, salt-sensitive hypertension
JMJD2A	Demethylation of H3K9me3, H3K4me3, and H3K27me3	Activate cardiac hypertrophy and alter cardiac gene expression
UTX (KDM6A)	Demethylation of H3K27	Regulate cardiac development
JMJD3	Demethylation of H3K27	Deficiency also leads to advanced atherosclerosis

### Histone Methylation of Key Genes in Cardiomyocytes and Blood Vessels

**Figure 2:**
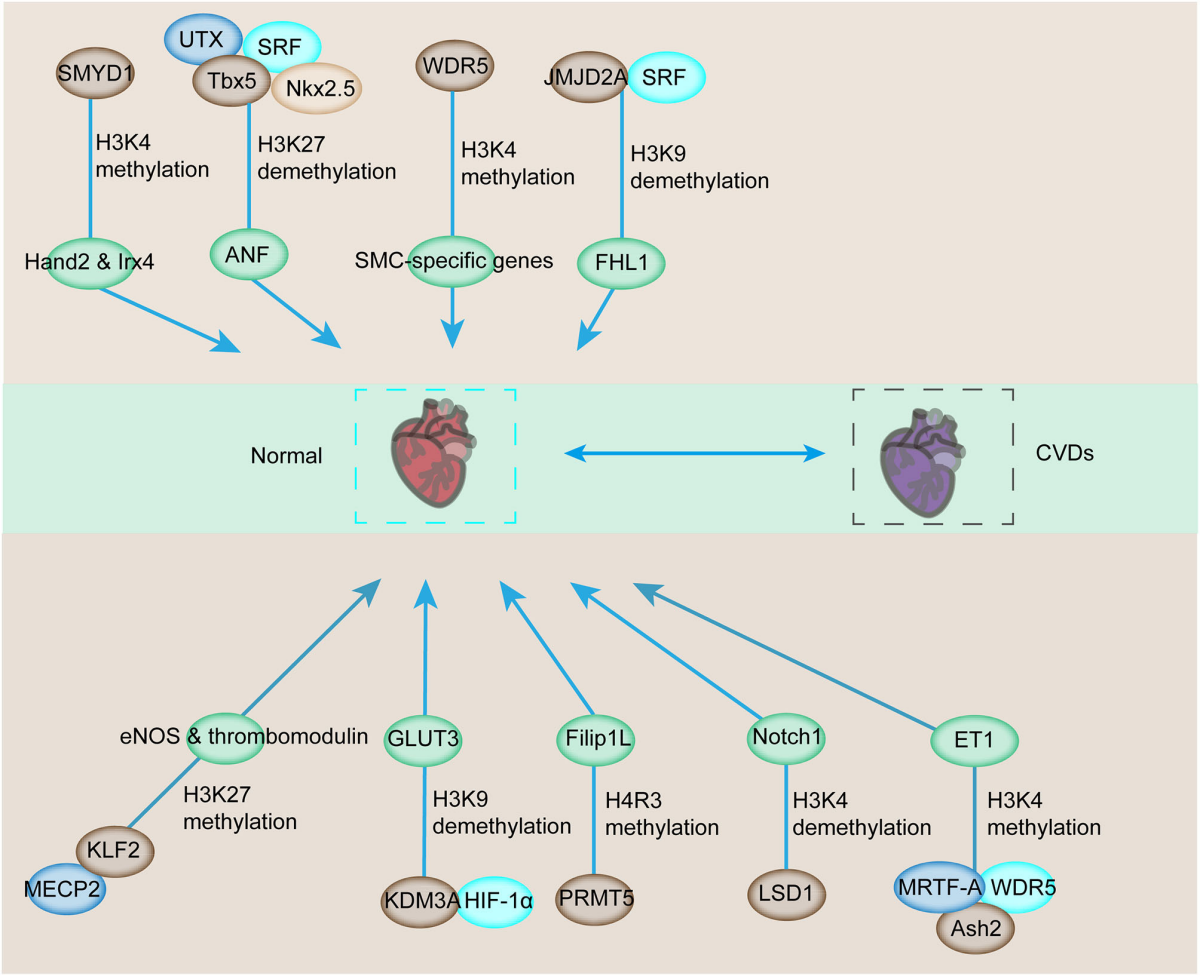
Downstream target genes involved in cardiovascular development and CVDs are affected by histone methylation and related enzymes. The downstream genes involved in the regulation of cardiovascular development can be modulated by histone methyltransferase or demethylase for transcriptional activation or repression. The abnormal activation or repression eventually contributes to the progression of CVDs.

Considering the close interaction among histone methyltransferases, demethylases, and the main regulators of muscle phenotype, the targeted cardiac genes are regulated by histone methylation (46, 63). A typical example of this interaction can be found in skeletal myocytes (SMCs). WDR5, a necessary component of the SET/MLL family of methyltransferases, regulates the expression of SMC-specific genes, including SM α-actin, SM22α, SM-MHC, and myocardia through the methylation of H3K4 on their corresponding promoters [Figure 2; (64)]. Ubiquitously transcribed tetratricopeptide repeat on chromosome X (UTX, a H3K27-specific histone demethylase), serum response factor (SRF), and other core cardiac transcription factors, such as Tbx5 and Nkx2.5, interact together. Their interaction synergistically modulates the expression of downstream genes, such as the atrial natriuretic factor (Figure 2). However, the inhibition of the UTX interaction between cardiac gene enhancers prevents cardiac differentiation [Figure 2; (65)]. In addition, increased histone acetylation and dimethylation are associated with increased expression of atrial natriuretic peptide and brain-type natriuretic peptide in the LV. Therefore, ubiquitously expressed histone methyltransferases and demethylases have regulatory roles in modulating the expression of genes involved in CVDs. The interactions between histone methyltransferases, demethylases, and transcriptional factors also affect the expression of genes exposed to various stimuli. JMJD2A, a histone demethylase, interacts with SRF/myocardia to elevate the level of four-and-a-half LIM domains 1 (FHL1), a cardiac hypertrophy biomechanical stress sensor when exposed to transverse aortic constriction (TAC, Figure 2). JMJD2A promotes cardiac hypertrophy. MRTFs regulate the expression of downstream genes via their interaction with methyltransferases and demethylases when exposed to stimuli. In ECs, MRTF-A interacts with Ash2 and WDR5, the components of COMPASS, and is recruited to the ET-1 promoter, exerting critical functions in vasoconstriction and endothelial dysfunction in CVDs in response to Ang II stimulation (66, 67). SMYD1-mediated histone methylation modulates the expression of *Hand2* and *Irx4*, which are essential cardiac transcription factors for RV formation [Figure 2; (68, 69)]. Histone demethylase JHDM2A deficiency modulates the PPARγ pathway via H3K9 modification (70). Therefore, demethylases and methyltransferases are involved in the recruitment and interaction with transcription factors that play a vital role in CVD pathologies.

In addition to cardiac genes, endothelial genes are also modulated by the combined regulation between transcription factors and histone methyltransferases and demethylases (71, 72). The interaction of epigenetic reader MECP2, H3K27 histone methyltransferase, enhancer of zeste homolog 2 (EZH2), and KLF2 triggers the inhibition of KLF2, which is a transcriptional factor responsible for the anti-inflammatory and antithrombotic surface via regulating numerous genes, including eNOS and thrombomodulin [Figure 2; (24)]. Additionally, the SMC phenotype switching in atherogenic conditions can be regulated by histone arginine methylation by targeting the transcription factor (73, 74). Protein arginine methyltransferase 4 mediates the upregulation of osteopontin through the dimethylation of R17 on histone H3, and this process promotes the recruitment of transcription factor USF1 (75, 76). The recruitment of USF1 is suppressed by arginine demethylase JMJD6 (77). Considering the sensitivity of ECs toward hypoxia, transcription factor interaction with epigenetic modification is also detected in hypoxia-induced upregulation of the glucose transporter, GLUT3, in ECs (78). The demethylase KDM3A is recruited to the transcriptional start site and enhancer regions of GLUT3 and facilitates the demethylation of H3K9 to induce GLUT3 expression in response to HIF1-α expression (78, 79). In addition, the interaction between HIF1-α and KDM3A has been confirmed by co-immunoprecipitation, and this process is inhibited by HIF1-α depletion (79). Thus, the interaction between HIF1-α and KDM3A modulates GLUT3 levels for the homeostasis of glucose levels, and this condition is required for maintaining energy supply under hypoxic conditions [Figure 2; (78)]. The demethylase LSD1 could serve as a repressor of Notch1, which specifically regulates cardiomyocyte proliferation within the trabeculae [Figure 2; (80)]. Based on these studies, histone methyltransferases and demethylases could modulate the expression of CVD-related genes by interacting with multiple transcription factors.

### Role of Histone Methylases and Demethylases in CVDs

**Figure 3:**
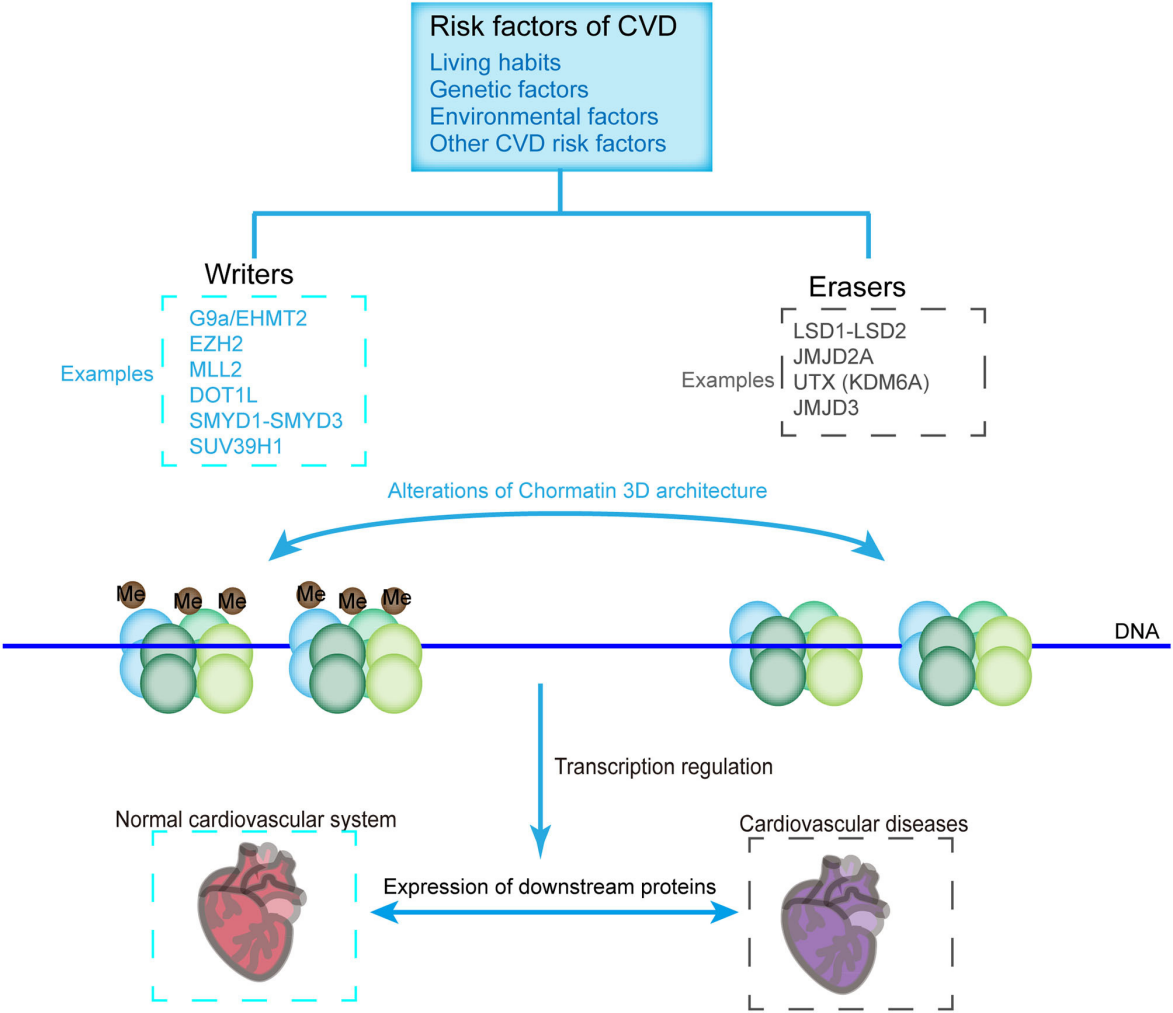
CVD-related histone methyltransferases and demethylases. Chromatin constitutes two structural states, namely, the methylated **(Left)** and demethylated **(Right)** states, which are catalyzed by methyltransferases and demethylases, respectively. Histone methylation state affects the loosening of the nucleosome, thus allowing the binding of transcriptional machineries or inhibiting the accessibility of the transcriptional machinery to genes, which are critical for the pathology of CVDs. Methyltransferases and demethylases can be affected by environmental factors, living habits, genetic factors, and other factors of CVDs.

Histone methylases such as G9a, EZH2, MLL2, DOT1L, SMYD1-SMYD3, and SUV39H1 and demethylases such as LSD1-LSD2, JMJD2A, UTX, and JMJD3 modulate the transcription of various cardiovascular genes and play an important role in cardiovascular development and CVDs. For example, G9a mediates H3K9 dimethylation and further suppresses the expression of cardiomyocyte-related genes (81). SMYD1 is a modulator of cardiac transcription factors for RV formation (68). H3K27me3, one of the most established histone modifiers, is modulated by EZH2, UTX, and JMJD3, and affects CVD progress [Figure 3; (45, 82–84)]. UTX interacts with SRF and other core cardiac transcription factors to affect heart development. The inhibition of UTX interaction also suppresses cardiac differentiation (65). Considering the vital importance of these methylase and demethylase in cardiac development and function, aberrant expression and mutation of the histone methylation modifiers, which can be affected by living habits, genetic factors, environmental factors, and other CVD risk factors, are critical in the pathology of CVDs (Figure 3).

### Histone Methylation in Atherosclerosis

EZH2 protects against cardiac pathology by inhibiting the expression of transcription factor Six1-a in cardiac progenitor cells (85). EZH2 plays a vital role in atherosclerosis (86). EZH2 overexpression leads to the development of atherosclerosis in ApoE^−/−^ mice by catalyzing the methylation of DNMT1-mediated ATP binding cassette transporter A1, thereby inhibiting macrophage cholesterol efflux and promoting foam cell formation (87). JMJD3 depletion in foam cells suppresses pro-fibrotic pathways, an important hallmark for atherosclerosis (38). Myeloid JMJD3 deficiency also leads to advanced atherosclerosis (88). Histone modification alterations, such as reduction of H3K9 and H3K27 methylation levels, have also been observed in patients with atherosclerotic plaques and carotid artery stenosis (20). Along with the progression of atherosclerosis, H3K4 methylation accumulates in SMCs; H3K9ac and H3K27ac are also enriched in atherosclerotic SMCs and macrophages, thus supporting the elevated HAT activity of GCN5-like protein 1 and HAT KAT8 (89). Additionally, H3K9ac accumulates in atherosclerotic plaques in ECs (90).

### Histone Methylation in Cardiac Hypertrophy

PRMT5 ameliorates cardiomyocyte hypertrophy and induces the methylation of H4R3me2 via the transcriptional activation of Filip1L and subsequent enhancement of β-catenin degradation [Figure 2; (63)]. PRMT5 deficiency contributes to the suppression of H4R3me2 and facilitates the progression of pathological cardiac hypertrophy (35). The depletion of muscle-specific SMYD1 (a H3K4 methyltransferase) leads to severe cardiac developmental defects [Figure 3; (91)]. Furthermore, in adult heart diseases, SMYD1 is elevated to restrict hypertrophic growth by directly repressing a group of hypertrophy-associated genes, including TGFβ3 and NPPA (92). The misregulation of PAX transactivation-domain interacting protein, a cofactor of H3K4 methylation, causes cardiac hypertrophy and failure (93). JMJD1C is involved in pathological cardiac hypertrophy, in which its expression level increases, and H3K9 methylation decreases during cardiac hypertrophy in humans and mice (94). JMJD1C contributes to hypertrophic cardiomyocytes stimulated with Ang II (95). In addition, cardiomyocyte remodeling occurs with the help of H3K9me3 methyltransferase, SUV39H1 upregulation and the H3K9me3 demethylases, JMJD downregulation [Figure 3; (96)]. As a H3K9me2 dimethyltransferase, EHMT1/2 protects mice from left ventricular hypertrophy (LVH) accompanied by increased global H3K9me2 levels induced by pressure overload (97). G9a mediates cardiomyocyte homeostasis by repressing genes involved in cardiomyocyte function, including anti-hypertrophic genes through its methylation on histone H3K9 and interaction with EZH2 and transcription factor myocyte-specific enhancer factor 2C (MEF2C) (81).

### Histone Methylation in Noonan Syndrome

The increased histone H3K4 methylation induced by haploinsufficiency of *RREB1* causes a Noonan-like RASopathy, which refers to the abnormal development in multiple part of the body including CVD, via SIN3A and KDM1A in human and murine cells (98). Moreover, disruption of the histone acetyltransferase MYST4 leads to a Noonan syndrome-like phenotype and hyperactivates MAPK signaling in humans and mice (99).

### Histone Methylation in Dilated Cardiomyopathy

The overexpression of Rae28, which is involved in the protein regulator of cytokinesis 1 (PRC1) complex in cardiomyocytes, leads to apoptosis of cardiomyocytes, irregular myofibrils, and dilated cardiomyopathy (100). By contrast, H3K79me3 is added by the histone-lysine N-methyltransferase DOT1L, which is repressed during dilated cardiomyopathy (101). DOT1L-specific depletion in cardiomyocytes triggers the total depletion of H3K79me2/3 and finally the reduction of the dystrophin (DMD) gene, a membrane-associated protein involved in dilated cardiomyopathy and muscular dystrophy (102). Consistently, DMD protein level is reduced in DOT1L-ablated hearts, which displays dilated cardiomyopathy (102). Similarly, the decrease of H3K9me2/3 and increase of H3K4me2 are correlated with dilated cardiomyopathy and accompanied by increased levels of myeloid/lymphoid or mixed-lineage leukemia protein 3 in the LV (64).

### Histone Methylation in Cardiac Development

H3K4 methyltransferase SMYD3 accumulates during the development of zebrafish heart, and SMYD3 knockdown results in severe defects, including pericardial edema and aberrant expression, of three heart-chamber markers in cardiac morphogenesis [Figure 3; (103)]. Therefore, histone methylation plays a critical function in the development of the heart, and its abnormal function leads to severe CVDs.

### Histone Methylation in Diabetic Cardiovascular Complications

Epigenetic modifications are critically involved in the long-lasting and detrimental effects of hyperglycemia on the cardiovascular system. Hyperglycemia induces aberrant changes in H3K4me2 and H3K9me2 in human monocytes. Monocytes from T2D patients exhibit SETD7-dependent epigenetic alterations (H3K4m) on NF-kB p65 promoter (104). Adverse epigenetic remodeling driven by SETD7 was associated with endothelial dysfunction and oxidative stress (105). The inhibitor of SETD7 alleviates the burden of CVD in patients with diabetes.

### Histone Methylation in Congenital Heart Defects

The mutations in epigenetic regulation are vital factors for the occurrence of congenital heart defects (106, 107). The decreases in heterochromatin H3K27me3 and its methyltransferase EZH2 are accompanied by Hutchinson–Gilford progeria syndrome, exhibiting atherosclerotic CVD phenotypes at an early age (108).

### Histone Methylation in Cardiac Ischemia/Reperfusion Injury

In response to cardiac ischemia/reperfusion (I/R) injury, histone, and methyltransferase G9a protein levels increased in caveolin knockout mice (109). The expression levels of MLL2 and G9a increased in advanced atherosclerosis compared with early atherosclerosis (37). Su(var)3–9 methyltransferase is associated with the pathogenesis of myocardial infarction (110). SUV39H1 deficiency or inhibition attenuates I/R-induced infarction and improves heart function in mice possibly by influencing reactive oxygen species (ROS) levels in a SIRT1-dependent manner (110). The mechanism underlying the epigenetic change in cardiac regulation needs to be elucidated to develop effective therapeutic strategies for CVDs.

### Combined Modulation of Histone Methylation and Acetylation in CVDs

Histone methylation and acetylation modification work together during the development of CVDs (46, 111, 112). For instance, nitric oxide (NO), which is produced by endothelial nitric oxide synthase (eNOS), is a major antiatherogenic factor in the blood vessel (113, 114). The activation of histone modifications, H3K9 and H4K12 acetylation, and H3K4 methylation are enriched at the proximal promoter site of NOS3 in ECs but not in SMCs, thus explaining the different expression patterns in ECs and SMCs (115). SMYD2 exhibits transcription repression on an SV40-luciferase reporter (116). The dimethylation of histone H3 lysine 36 by SMYD2 is accompanied by its interaction with Sin3, a HDAC1-containing complex, implying orchestrated regulation of methylation and acetylation [Figure 3; (117)].

### Histone Methylation-Mediated Cellular Functions and Pathways in CVDs

Histone modification affects many cellular pathways essential for the normal function and development of the heart and blood vessels (110, 118–120). The methyltransferase SET7 induces the upregulation of NF-κB p65 as a result of enhanced monomethylation of H3K4 in aortic ECs (121). SET7 can also be mediated by transient hyperglycemia, triggering H3K4me1 and further activating NF-κB p65 and NF-κB-dependent inflammatory genes in ECs, thus suggesting its critical role in hyperglycemia-mediated vascular complications (105, 122). SET7 may act as a promising target for the prevention of atherosclerotic vascular disease in patients with cardiometabolic disturbances (122, 123). In addition, H3K4me1 is correlated with the expression of oxidant genes (iNOS and COX-2) and elevated plasma levels of ICAM-1 and MCP-1 (124). EZH2 ablation or enzymatic inactivation in the fetal heart decreases cardiomyocyte proliferation and increases apoptosis and lethal congenital malformations (85, 86). Although the function of the paralog gene EZH1 can be disregarded during early cardiac development, this function is essential for neonatal heart regeneration (125). EZH1 overexpression leads to cardiac regeneration in 10-day-old mice, which usually have non-regenerative heart (126). MLL2, a methyltransferase that is widely expressed in adult tissues, functions in embryonic development (127–132). As a H3K36-specific methyltransferase, HYPB (also known as SETD2 and KMT3A) homozygous disruption leads to embryonic lethality at E10.5–E11.5 caused by severe vascular defects in the embryo, yolk sac, and placenta (133). DOT1L catalyzes the methylation of histone H3K79 and modulates transcriptional elongation, cell cycle progression, somatic reprogramming, development, and DNA damage repair (134–138).

## Therapeutic Potential of Epigenetic Inhibitors As Cardiovascular Drugs

Considering that epigenetic modification plays an important role in the progression of CVDs, small-molecule epigenetic drugs against CVDs should be developed. The reversible nature of epigenetic modifications allows the modulation and restoration of phenotypes via some inhibitors or dietary restrictions (139–142). In comparison with the other types of epigenetic inhibitors, the inhibitors of histone methylation have not been extensively researched and remain an undeveloped source of pharmacological interventions.

**Figure 4:**
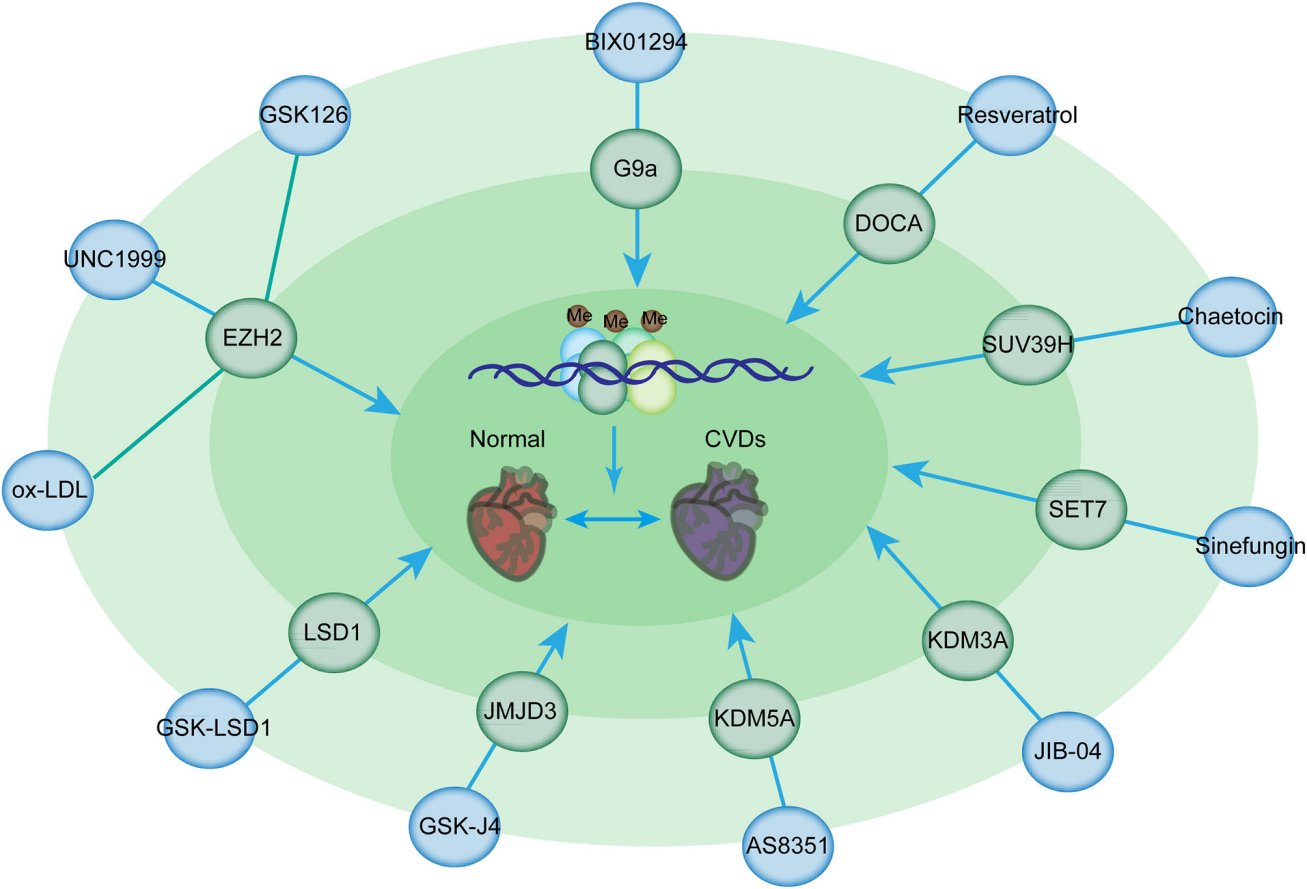
Histone methylation-modifying inhibitors and their targets for the prevention of CVDs. Histone methylation-modifying inhibitors are indicated in bold together with their respective targets. Each compound specifically targets the enzymes involved in methylation regulation, thus affecting chromatin accessibility and binding of transcription factors critical for CVDs. These alterations modulate the expression of downstream genes in CVDs.

Among these inhibitors, GSK126 is a potent and highly selective methyltransferase inhibitor for the histone methyltransferase EZH2 [Figure 4; (143)]. Given that myeloid EZH2 deficiency in mice leads to improvement in chronic inflammatory disorders such as CVDs, GSK126 has been used to reduce macrophage pro-inflammatory responses (143). Moreover, EZH2 plays an important role in atherosclerosis. EZH2 induces lipid accumulation when stimulated with ox-LDL and macrophage activation and inflammation in THP-1- and RAW264.7-derived macrophages (144). The overexpression of EZH2 in mice can augment the atherosclerosis plaque size by repressing the expression of Abga1/Abcg1 (145). Therefore, GSK126 has a potential therapeutic effect of GSK126 in atherosclerosis treatment. Notably, statins can reduce EZH2 expression levels in ECs, suggesting that they can serve as the potential therapeutic target in atherosclerosis treatment (145, 146). Additionally, the inhibition of EZH2 by UNC1999 significantly inhibits VSMC proliferation induced by PDGF-BB and neointima formation caused by wire-guided common carotid injury, mediated by the enhanced transcription of the cyclin-dependent kinase inhibitor p16INK4A [Figure 4; (147)]. Inhibition of EZH2 activity by its inhibitor, UNC1999, or knockdown of EZH2 by its shRNA, leads to VSMC loss, while overexpression of EZH2 facilitates VSMC growth, therefore promoting a tear in the inner layer of the aortic wall, which allows blood to enter into the wall of the aorta, as evidenced by fragmentation of elastic fibers and VSMC loss (148).

G9a is responsible for the homeostasis of cardiomyocytes by mediating H3K9 dimethylation to inhibit the expression of cardiomyocyte-related genes and the formation of a complex with EZH2 and MEF2C (149). TAC mice, which were administered with BIX-01294 (a G9a inhibitor), had improved cardiac function and prevented the development of hypertrophy (150). BIX01294 promotes the expansion of adult cardiac progenitor cells without changing their phenotype or differentiation potential, suggesting that this drug can be used to generate large numbers of native cardiac progenitor cells for the treatment of cardiac disease (150). Furthermore, EPZ005687, a selective inhibitor of Ezh2, significantly inhibits the progression of pulmonary arterial hypertension induced by TAC (151). Resveratrol is beneficial for deoxycorticosterone acetate salt-induced hypertension, a risk factor for cardiac disease, partially by suppressing H3K27 methylation in the blood vessels (152). Additionally, Su(var)3–9 methyltransferase is associated with the pathogenesis of myocardial infarction (110). SUV39H is upregulated in neonatal rat ventricular myocytes in cardiac ischemia/reperfusion injury (114). Chaetocin is a promising epigenetic inhibitor for H3K9 methyltransferase SUV39H (153). The administration of chaetocin preserved changes in histone methylation and improved survival in a rat model of high-salt-diet-induced heart failure, suggesting the beneficial effects of methyltransferase inhibitors for the treatment of heart disease (154). Furthermore, the intraperitoneal administration of chaetocin improves survival and decreases infarct size in C57/BL6 mice following myocardial infarction (155). Chaetocin therapy also suppresses the expression of MMP9, which is responsible for the destabilization of plaque (156). Thus, further investigations are needed to determine the potential use of this compound in CVDs. Sinefungin, a SET7 inhibitor inhibits the heightened production of TNFα and IL-6 in a dose-dependent manner following stimulation with LPS in an atherosclerotic disease mouse model (157).

KDM3A, a specific H3K9me2 demethylase, results in LVH and fibrosis induced by pressure overload (158). KDM3A promotes TAC-induced hypertrophic remodeling *in vivo* (158). JIB-04, a pan KDM inhibitor, prevents pressure overload-induced LVH and fibrosis (159, 160). JIB-04 inhibits KDM3A and the expression of proteins involved in myocardial fibrosis (159). It also protects mice against I/R injury (160). AS8351 is a KDM5B inhibitor that can induce and sustain active chromatin marks to facilitate the induction of cardiomyocyte-like cells (161). JMJD3 plays a pivotal role in hypertrophy (162). The overexpression of JMJD3 promotes cardiomyocyte hypertrophy; JMJD3 silencing or the administration of GSK-J4 (its inhibitor) suppresses ISO-induced cardiac hypertrophy (163). Another example showed IOX1, a JMJD2A inhibitor, suppressed the proliferation and migration of VSMCs induced by angiotensin II by regulating the expression of cell cycle-related proteins and can therefore serve as a potential therapeutic agent in the treatment of atherosclerosis (164). In addition, the inhibition of LSD1 with GSK-LSD1 in mice prevents the development of fibrosis, an EMT-mediated process, in the heart and dilatation, thus preventing heart failure (165).

Although the critical functions of histone PTMs in CVDs have been revealed, much work is needed to comprehensively illustrate the function of these proteins in various processes and their utilization in therapeutic applications. Currently, no epigenetically active agents have entered clinical trials for CVDs. Further investigations on the potential use of epigenetically active compounds are urgently needed for the treatment of CVDs.

## Conclusions and Future Perspectives

Epigenetic modifications, such as DNA methylation, histone methylation, and acetylation, are promising therapeutic strategies for the treatment of CVDs (166–170). Despite the recent advances in epigenetic modifications in CVDs, the potential epigenetic inhibitors for CVD therapy have not been identified. Furthermore, a better understanding of the mechanism of epigenetic modification that regulates CVD progression is urgently needed to develop new strategies for the treatment of CVDs. Further studies are needed to improve the pharmacology of these potential inhibitors, because a non-specific inhibitor would cause unnecessary suppression or activation of a set of genes, causing adverse outcomes. Considering the high resemblance in the modifications on different histone proteins, the design of a highly selective inhibitor that targets a particular protein remains a challenge. Building on the foundation of currently available knowledge will help us to take full advantage of the incredible therapeutic capacity of epigenetic drugs.

Considering the complexity of the pathogenesis of CVDs, the important role of epigenetics, especially histone methylation, should be determined. In general, histone methylation mainly regulates the transcription of downstream genes that are closely related to cardiovascular development or affect the activity of related signaling pathways. Histone methylation can also cooperate with acetylation and other modifications to precisely regulate gene transcription. A deep understanding of the related processes will help us to clarify the regulatory mechanism of cardiovascular development and the pathogenesis of CVDs. It can also provide a theoretical basis for the next step of screening important therapeutic targets and developing related inhibitors.

With the use histone methyltransferase or demethylase inhibitors for CVD treatment and intervention, we should still focus on the various abilities of these inhibitors to activate or inhibit multiple gene transcription, causing complex, and potential side effects of related inhibitors. We should use transcriptomics and proteomics to analyze their pharmacological mechanism carefully to achieve the best therapeutic effect. Another important problem is that the inhibitors of histone methyltransferases and demethylases generally lack specificity. The next important task is the design of specific inhibitors for a certain enzyme based on different methyltransferases or demethylases by using specific three-dimensional structural analysis methods and combined with pharmacological approaches. The best therapeutic effect on CVDs can be achieved by precisely regulating the histone methylation or demethylation of a specific site.

## Author Contributions

YiL and YY conceptualized and wrote the manuscript and created Figures. YY and YingL contributed to the writing of the manuscript. YY and R-XY reviewed and modified the manuscript. All authors approved the final version of the manuscript.

## Funding

This work was supported by the National Natural Science Foundation of China (No. 31900502), and the Henan Medical Science and Technology Joint Building Program (Nos. LHGJ20190236 and LHGJ20190223).

## Conflict of Interest

The authors declare that the research was conducted in the absence of any commercial or financial relationships that could be construed as a potential conflict of interest.

## Publisher's Note

All claims expressed in this article are solely those of the authors and do not necessarily represent those of their affiliated organizations, or those of the publisher, the editors and the reviewers. Any product that may be evaluated in this article, or claim that may be made by its manufacturer, is not guaranteed or endorsed by the publisher.

## Abbreviations

CVD: cardiovascular diseases
PTMs: post-translational modifications
LV: left ventricle
RV: right ventricle
HAT: histone acetyltransferase
HDAC: histone deacetylases
KMT: histone lysine methyltransferase
LSD: lysine-specific demethylase
HDM: histone demethylase
JHDM: Jumonji C-domain-containing family
SMYD: SET domain and MYND domain protein
JARID: Jumonji, and AT rich interactive domain 1D
FHL1: four-and-a-half LIM domains 1
TAC: transverse aortic constriction
EZH2: enhancer of zeste homolog 2
SMC: skeletal myocyte
UTX: ubiquitously transcribed tetratricopeptide repeat on chromosome X
LVH: left ventricular hypertrophy
MEF2C: myocyte-specific enhancer factor 2C
DMD: Duchenne muscular dystrophy
ROS: reactive oxygen species
NO: nitric oxide
eNOS: endothelial nitric oxide synthase
EC: endothelia cell
NF-κB: nuclear factor kappa-B
VSMC: vascular smooth muscle cell
SET: Su(var)3–9, Enhancer of zeste and Trithorax
MLL: mixed lineage leukemia protein
SETD7: SET domain containing 7
EHMT2/G9a: euchromatic histone-lysine N-methyltransferase 2
PRMT: protein arginine methyltransferase
DOT1L: DOT1 like histone lysine methyltransferase
WDR5: WD repeat-containing protein 5
KLF2: Krüppel-like factor 2
JMJD: Jumonji C domain-containing.

## References

[1] Chapman N McWhirter RE Jose KA Schultz MG Ezzy D Nelson MR. General practitioners maintain a focus on blood pressure management rather than absolute cardiovascular disease risk management. J Eval Clin Pract. (2021). 10.1111/jep.13569. [Epub ahead of print]. 33851486

[2] Kullawong N Apidechkul T Upala P Tamornpark R Keawdounglek V Wongfu C. Factors associated with elevated low-density lipoprotein cholesterol levels among hill tribe people aged 30 years and over in Thailand: a cross-sectional study. BMC Public Health. (2021) 21:498. 10.1186/s12889-021-10577-3 33711970 PMC7953743

[3] Berg DD Freedman BL Bonaca MP Jarolim P Scirica BM Goodrich EL. Cardiovascular biomarkers and heart failure risk in stable patients with atherothrombotic disease: a nested biomarker study from TRA 2°P-TIMI 50. J Am Heart Assoc. (2021) 10:e018673. 10.1161/JAHA.120.018673 33884889 PMC8200769

[4] Wang J Liu F Li J Huang K Yang X Chen J. Fruit and vegetable consumption, cardiovascular disease, and all-cause mortality in China. Sci China Life Sci. (2021). 10.1007/s11427-020-1896-x. [Epub ahead of print]. 33893978

[5] Mazzolai L Alatri A Riviere AB De Carlo M Heiss C Espinola-Klein C. Progress in aorta and peripheral cardiovascular disease research. Cardiovasc Res. (2021) 117:2045–53. 10.1093/cvr/cvab144 33892507 PMC8600478

[6] Hayen A Glasziou PP Doust JA. Coronary artery calcium scoring in cardiovascular risk assessment of people with family histories of early onset coronary artery disease. Med J Austral. (2021) 214:440.e1. 10.5694/mja2.51037 33887798

[7] Grant JK Ebner B Vincent L Maning J Olorunfemi O Olarte NI. Assessing in-hospital cardiovascular, thrombotic and bleeding outcomes in patients with chronic liver disease undergoing left ventricular assist device implantation. Thromb Res. (2021) 202:184–90. 10.1016/j.thromres.2021.04.010 33892219

[8] Li L Zhao M Wang C Zhang S Yun C Chen S. Early onset of hyperuricemia is associated with increased cardiovascular disease and mortality risk. Clin Res Cardiol. (2021) 10:1096–105. 10.1007/s00392-021-01849-4 33846840

[9] Wahid A Chen W Wang X Tang X. High-mobility group box 1 serves as an inflammation driver of cardiovascular disease. Biomed Pharmacother. (2021) 139:111555. 10.1016/j.biopha.2021.111555 33865014

[10] Salazar-Tortosa DF Pascual-Gamarra JM Labayen I Ruperez AI Censi L Beghin L. Interplay of physical activity and genetic variants of the endothelial lipase on cardiovascular disease risk factors. Pediatr Res. (2021). 10.1038/s41390-021-01519-1. [Epub ahead of print]. 33859368

[11] Shih CC Shih YL Chen JY. The association between homocysteine levels and cardiovascular disease risk among middle-aged and elderly adults in Taiwan. BMC Cardiovasc Disord. (2021) 21:191. 10.1186/s12872-021-02000-x 33879044 PMC8056530

[12] Nealand B Wu J. Sodium, blood pressure, and the likely massive avoidable burden of cardiovascular disease. Circulation. (2021) 143:1568–70. 10.1161/CIRCULATIONAHA.120.052654 33872077

[13] Lee MT Mahtta D Ramsey DJ Liu J Misra A Nasir K. Sex-related disparities in cardiovascular health care among patients with premature atherosclerotic cardiovascular disease. JAMA Cardiol. (2021). 10.1001/jamacardio.2021.0683. [Epub ahead of print]. PMC8060887 33881448

[14] Ammous F Zhao W Ratliff SM Mosley TH Bielak LF Zhou X. Epigenetic age acceleration is associated with cardiometabolic risk factors and clinical cardiovascular disease risk scores in African Americans. Clin Epigenet. (2021) 13:55. 10.1186/s13148-021-01035-3 33726838 PMC7962278

[15] Pons D de Vries FR van den Elsen PJ Heijmans BT Quax PH Jukema JW. Epigenetic histone acetylation modifiers in vascular remodelling: new targets for therapy in cardiovascular disease. Eur Heart J. (2009) 30:266–77. 10.1093/eurheartj/ehn603 19147603

[16] Handy DE Castro R Loscalzo J. Epigenetic modifications: basic mechanisms and role in cardiovascular disease. Circulation. (2011) 123:2145–56. 10.1161/CIRCULATIONAHA.110.956839 21576679 PMC3107542

[17] Zawada AM Rogacev KS Heine GH. Clinical relevance of epigenetic dysregulation in chronic kidney disease-associated cardiovascular disease. Nephrol Dialysis Transpl. (2013) 28:1663–71. 10.1093/ndt/gft042 23512108

[18] Zaiouand M Bakillah A. Epigenetic regulation of ATP-binding cassette protein A1 (ABCA1) gene expression: a new era to alleviate atherosclerotic cardiovascular disease. Diseases. (2018) 6:34. 10.3390/diseases6020034 29751497 PMC6023542

[19] Vinci MC Polvani G Pesce M. Epigenetic programming and risk: the birthplace of cardiovascular disease? Stem Cell Rev Rep. (2013) 9:241–53. 10.1007/s12015-012-9398-z 22773406

[20] Schiano C Vietri MT Grimaldi V Picascia A De Pascale MR Napoli C. Epigenetic-related therapeutic challenges in cardiovascular disease. Trends Pharmacol Sci. (2015) 36:226–35. 10.1016/j.tips.2015.02.005 25758254

[21] Zhang L Tian S Pei M Zhao M Wang L Jiang Y. Crosstalk between histone modification and DNA methylation orchestrates the epigenetic regulation of the costimulatory factors, Tim3 and galectin9, in cervical cancer. Oncol Rep. (2019) 42:2655–69. 10.3892/or.2019.7388 31661141 PMC6859457

[22] Qadirand MI Anwer F. Epigenetic modification related to acetylation of histone and methylation of DNA as a key player in immunological disorders. Crit Rev Eukaryot Gene Expr. (2019) 29:1–15. 10.1615/CritRevEukaryotGeneExpr.2018024760 31002589

[23] Rizzacasa B Amati F Romeo F Novelli G Mehta JL. Epigenetic modification in coronary atherosclerosis: JACC review topic of the week. J Am Coll Cardiol. (2019) 74:1352–65. 10.1016/j.jacc.2019.07.043 31488273

[24] Buck JM O'Neill HC Stitzel JA. Developmental nicotine exposure engenders intergenerational downregulation and aberrant posttranslational modification of cardinal epigenetic factors in the frontal cortices, striata, and hippocampi of adolescent mice. Epigenet Chromatin. (2020) 13:13. 10.1186/s13072-020-00332-0 32138755 PMC7059320

[25] Marchione AD Thompson Z Kathrein KL. DNA methylation and histone modifications are essential for regulation of stem cell formation and differentiation in zebrafish development. Brief Funct Genomics. (2021) elab022. 10.1093/bfgp/elab022. [Epub ahead of print]. 33782688

[26] Lorzadeh A Romero-Wolf M Goel A Jadhav U. Epigenetic regulation of intestinal stem cells and disease: a balancing act of DNA and histone methylation. Gastroenterology. (2021) 160:2267–82. 10.1053/j.gastro.2021.03.036 33775639 PMC8169626

[27] Huang LY Hsu DW Pears CJ. Methylation-directed acetylation of histone H3 regulates developmental sensitivity to histone deacetylase inhibition. Nucleic Acids Res. (2021) 49:3781–95. 10.1093/nar/gkab154 33721015 PMC8053100

[28] Honma K Machida C Mochizuki K Goda T. Glucose and TNF enhance expression of TNF and IL1B, and histone H3 acetylation and K4/K36 methylation, in juvenile macrophage cells. Gene X. (2020) 5:100034. 10.1016/j.gene.2020.100034 32550560 PMC7285958

[29] Zhang T Du E Liu Y Cheng J Zhang Z Xu Y. Anticancer effects of zinc oxide nanoparticles through altering the methylation status of histone on bladder cancer cells. Int J Nanomed. (2020) 15:1457–68. 10.2147/IJN.S228839 32184598 PMC7062395

[30] Zhou H Liu Y Liang Y Zhou D Li S Lin S. The function of histone lysine methylation related SET domain group proteins in plants. Protein Sci. (2020) 29:1120–37. 10.1002/pro.3849 32134523 PMC7184775

[31] Raiymbek G An S Khurana N Gopinath S Larkin A Biswas S. An H3K9 methylation-dependent protein interaction regulates the non-enzymatic functions of a putative histone demethylase. eLife. (2020) 9:e53155. 10.7554/eLife.53155.sa2 32195666 PMC7192584

[32] Kronfol MM Jahr FM Dozmorov MG Phansalkar PS Xie LY Aberg KA. DNA methylation and histone acetylation changes to cytochrome P450 2E1 regulation in normal aging and impact on rates of drug metabolism in the liver. GeroScience. (2020) 42:819–32. 10.1007/s11357-020-00181-5 32221779 PMC7287002

[33] Lorton BM Harijan RK Burgos ES Bonanno JB Almo SC Shechter D. A binary arginine methylation switch on histone H3 arginine 2 regulates its interaction with WDR5. Biochemistry. (2020) 59:3696–708. 10.1021/acs.biochem.0c00035 32207970 PMC7529705

[34] Yang M Lin X Segers F Suganthan R Hildrestrand GA Rinholm JE. OXR1A, a coactivator of PRMT5 regulating histone arginine methylation. Cell Rep. (2020) 30:4165–78.e7. 10.1016/j.celrep.2020.02.063 32209476

[35] Beacon TH Xu W Davie JR. Genomic landscape of transcriptionally active histone arginine methylation marks, H3R2me2s and H4R3me2a, relative to nucleosome depleted regions. Gene. (2020) 742:144593. 10.1016/j.gene.2020.144593 32199949

[36] Vallianatos CN Raines B Porter RS Bonefas KM Wu MC Garay PM. Mutually suppressive roles of KMT2A and KDM5C in behaviour, neuronal structure, and histone H3K4 methylation. Commun Biol. (2020) 3:278. 10.1038/s42003-020-1001-6 32483278 PMC7264178

[37] Douillet D Sze CC Ryan C Piunti A Shah AP Ugarenko M. Uncoupling histone H3K4 trimethylation from developmental gene expression via an equilibrium of COMPASS, Polycomb and DNA methylation. Nat Genet. (2020) 52:615–25. 10.1038/s41588-020-0618-1 32393859 PMC7790509

[38] Imuta H Fujita D Oba S Kiyosue A Nishimatsu H Yudo K. Histone methylation and demethylation are implicated in the transient and sustained activation of the interleukin-1β gene in murine macrophages. Heart Vessels. (2020) 35:1746–54. 10.1007/s00380-020-01670-5 32676696

[39] Healton SE Pinto HD Mishra LN Hamilton GA Wheat JC Swist-Rosowska K. H1 linker histones silence repetitive elements by promoting both histone H3K9 methylation and chromatin compaction. Proc Natl Acad Sci USA. (2020) 117:14251–8. 10.1073/pnas.1920725117 32513732 PMC7322038

[40] Singh SK Bahal R Rasmussen TP. Evidence that miR-152-3p is a positive regulator of SETDB1-mediated H3K9 histone methylation and serves as a toggle between histone and DNA methylation. Exp Cell Res. (2020) 395:112216. 10.1016/j.yexcr.2020.112216 32768498

[41] Li J Qiu Y Li L Wang J Cheuk YC Sang R. Histone methylation inhibitor DZNep ameliorated the renal ischemia-reperfusion injury via inhibiting TIM-1 mediated T cell activation. Front Med. (2020) 7:305. 10.3389/fmed.2020.00305 32754604 PMC7365856

[42] Cusack M King HW Spingardi P Kessler BM Klose RJ Kriaucionis S. Distinct contributions of DNA methylation and histone acetylation to the genomic occupancy of transcription factors. Genome Res. (2020) 30:1393–406. 10.1101/gr.257576.119 32963030 PMC7605266

[43] Zhou S Feng S Qin W Wang X Tang Y Yuan S. Epigenetic regulation of spermatogonial stem cell homeostasis: from DNA methylation to histone modification. Stem Cell Rev Rep. (2021) 17:562–80. 10.1007/s12015-020-10044-3 32939648

[44] Grigore F Yang H Hanson ND VanBrocklin MW Sarver AL Robinson JP. BRAF inhibition in melanoma is associated with the dysregulation of histone methylation and histone methyltransferases. Neoplasia. (2020) 22:376–89. 10.1016/j.neo.2020.06.006 32629178 PMC7338995

[45] Li Z Jiang G Liu X Ding X Zhang D Wang X. Histone demethylase SlJMJ6 promotes fruit ripening by removing H3K27 methylation of ripening-related genes in tomato. New Phytol. (2020) 227:1138–56. 10.1111/nph.16590 32255501

[46] Raveendran VV Al-Haffar K Kunhi M Belhaj K Al-Habeeb W Al-Buraiki J. Protein arginine methyltransferase 6 mediates cardiac hypertrophy by differential regulation of histone H3 arginine methylation. Heliyon. (2020) 6:e03864. 10.1016/j.heliyon.2020.e03864 32420474 PMC7218648

[47] Ciesielski O Biesiekierska M Balcerczyk A. Epigallocatechin-3-gallate (EGCG) alters histone acetylation and methylation and impacts chromatin architecture profile in human endothelial cells. Molecules. (2020) 25:2326. 10.3390/molecules25102326 32429384 PMC7287656

[48] Sousa LO Sobral LM de Almeida LO Garcia CB Greene LJ Leopoldino AM. SET protein modulates H4 histone methylation status and regulates miR-137 level in oral squamous cell carcinoma. Epigenomics. (2020) 12:475–85. 10.2217/epi-2019-0181 32267167

[49] Sun P Zhang SJ Maksim S Yao YF Liu HM Du J. Epigenetic modification in macrophages: a promising target for tumor and inflammation-associated disease therapy. Curr Top Med Chem. (2019) 19:1350–62. 10.2174/1568026619666190619143706 31215380

[50] Dhall A Shelton PMM Delachat AM Leonen CJA Fierz B Chatterjee C. Nucleosome binding by the lysine specific demethylase 1 (LSD1) enzyme enables histone H3 demethylation. Biochemistry. (2020) 59:2479–83. 10.1021/acs.biochem.0c00412 32567837 PMC7640899

[51] Souto JA Sarno F Nebbioso A Papulino C Alvarez R Lombino J. A new family of Jumonji C domain-containing KDM inhibitors inspired by natural product purpurogallin. Front Chem. (2020) 8:312. 10.3389/fchem.2020.00312 32523934 PMC7261929

[52] Ambrosio S Ballabio A Majello B. Histone methyl-transferases and demethylases in the autophagy regulatory network: the emerging role of KDM1A/LSD1 demethylase. Autophagy. (2019) 15:187–96. 10.1080/15548627.2018.1520546 30208749 PMC6333462

[53] Nagasaka M Tsuzuki K Ozeki Y Tokugawa M Ohoka N Inoue Y. Lysine-Specific demethylase 1 (LSD1/KDM1A) is a novel target gene of c-Myc. Biol Pharmaceut Bull. (2019) 42:481–8. 10.1248/bpb.b18-00892 30828079

[54] Sugeedha J Gautam J Tyagi S. SET1/MLL family of proteins: functions beyond histone methylation. Epigenetics. (2021) 16:469–87. 10.1080/15592294.2020.1809873 32795105 PMC8078731

[55] Majello B Gorini F Sacca CD Amente S. Expanding the role of the histone lysine-specific demethylase LSD1 in cancer. Cancers. (2019) 11:324. 10.3390/cancers11030324 30866496 PMC6468368

[56] Liu H Pattie P Chandrasekara S Spencer A Dear AE. Epigenetic regulation of miRNA-124 and multiple downstream targets is associated with treatment response in myeloid malignancies. Oncol Lett. (2016) 12:2175–80. 10.3892/ol.2016.4912 27602159 PMC4998575

[57] Zhao Q Li S Li N Yang X Ma S Yang A. miR-34a targets HDAC1-regulated H3K9 acetylation on lipid accumulation induced by homocysteine in foam cells. J Cell Biochem. (2017) 118:4617–27. 10.1002/jcb.26126 28485501

[58] Guo Z Li Z Liu Y An Z Peng M Shen WH. MRG1/2 histone methylation readers and HD2C histone deacetylase associate in repression of the florigen gene FT to set a proper flowering time in response to day-length changes. New Phytol. (2020) 227:1453–66. 10.1111/nph.16616 32315442

[59] Rajan PK Udoh UA Sanabria JD Banerjee M Smith G Schade MS. The role of histone acetylation-/methylation-mediated apoptotic gene regulation in hepatocellular carcinoma. Int J Mol Sci. (2020) 21:8894. 10.3390/ijms21238894 33255318 PMC7727670

[60] Fallah MS Szarics D Robson CM Eubanks JH. Impaired regulation of histone methylation and acetylation underlies specific neurodevelopmental disorders. Front Genet. (2020) 11:613098. 10.3389/fgene.2020.613098 33488679 PMC7820808

[61] Bai L Sun H Jiang W Yang L Liu G Zhao X. DNA methylation and histone acetylation are involved in Wnt10b expression during the secondary hair follicle cycle in Angora rabbits. J Anim Physiol Anim Nutr. (2021) 105:599–609. 10.1111/jpn.13481 33404138

[62] Li H Wen Y Wu S Chen D Luo X Xu R. Epigenetic modification of enhancer of zeste homolog 2 modulates the activation of dendritic cells in allergen immunotherapy. Int Arch Allergy Immunol. (2019) 180:120–7. 10.1159/000500882 31256157

[63] Cai S Wang P Xie T Li Z Li J Lan R. Histone H4R3 symmetric di-methylation by Prmt5 protects against cardiac hypertrophy via regulation of Filip1L/beta-catenin. Pharmacol Res. (2020) 161:105104. 10.1016/j.phrs.2020.105104 32739429

[64] Alicea-Velazquez NL Shinsky SA Loh DM Lee JH Skalnik DG Cosgrove MS. targeted disruption of the interaction between WD-40 repeat protein 5 (WDR5) and mixed lineage leukemia (MLL)/SET1 family proteins specifically inhibits MLL1 and SETd1A methyltransferase complexes. J Biol Chem. (2016) 291:22357–72. 10.1074/jbc.M116.752626 27563068 PMC5077178

[65] Lee S Lee JW Lee SK. UTX, a histone H3-lysine 27 demethylase, acts as a critical switch to activate the cardiac developmental program. Dev Cell. (2012) 22:25–37. 10.1016/j.devcel.2011.11.009 22192413 PMC4111644

[66] Karatas H Townsend EC Bernard D Dou Y Wang S. Analysis of the binding of mixed lineage leukemia 1 (MLL1) and histone 3 peptides to WD repeat domain 5 (WDR5) for the design of inhibitors of the MLL1-WDR5 interaction. J Med Chem. (2010) 53:5179–85. 10.1021/jm100139b 20575550 PMC4289617

[67] Shimoda H Doi S Nakashima A Sasaki K Doi T Masaki T. Inhibition of the H3K4 methyltransferase MLL1/WDR5 complex attenuates renal senescence in ischemia reperfusion mice by reduction of p16(INK4a). Kidney Int. (2019) 96:1162–75. 10.1016/j.kint.2019.06.021 31570196

[68] Wang Z Schwartz RJ Liu J Sun F Li Q Ma Y. Smyd1 orchestrates early heart development through positive and negative gene regulation. Front Cell Dev Biol. (2021) 9:654682. 10.3389/fcell.2021.654682 33869215 PMC8047137

[69] Chow MZ Sadrian SN Keung W Geng L Ren L Kong CW. Modulation of chromatin remodeling proteins SMYD1 and SMARCD1 promotes contractile function of human pluripotent stem cell-derived ventricular cardiomyocyte in 3D-engineered cardiac tissues. Sci Rep. (2019) 9:7502. 10.1038/s41598-019-42953-w 31097748 PMC6522495

[70] Li Y He J Sui S Hu X Zhao Y Li N. Clenbuterol upregulates histone demethylase JHDM2a via the beta2-adrenoceptor/cAMP/PKA/p-CREB signaling pathway. Cell Signal. (2012) 24:2297–306. 10.1016/j.cellsig.2012.07.010 22820505

[71] Ye X Qian Y Wang Q Yuan W Mo X Li Y. SMYD1, an SRF-interacting partner, is involved in angiogenesis. PLoS ONE. (2016) 11:e0146468. 10.1371/journal.pone.0146468 26799706 PMC4723226

[72] Wojtala M Dabek A Rybaczek D Sliwinska A Swiderska E Slapek K. Silencing lysine-specific histone demethylase 1 (LSD1) causes increased HP1-positive chromatin, stimulation of DNA repair processes, and dysregulation of proliferation by Chk1 phosphorylation in human endothelial cells. Cells. (2019) 8:1212. 10.3390/cells8101212 31591366 PMC6829479

[73] Zhang BF Jiang H Chen J Guo X Hu Q Yang S. KDM3A inhibition attenuates high concentration insulininduced vascular smooth muscle cell injury by suppressing MAPK/NFkappaB pathways. Int J Mol Med. (2018) 41:1265–74. 10.3892/ijmm.2017.3351 29286083 PMC5819917

[74] Zhang C Ge S Gong W Xu J Guo Z Liu Z. LncRNA ANRIL acts as a modular scaffold of WDR5 and HDAC3 complexes and promotes alteration of the vascular smooth muscle cell phenotype. Cell Death Dis. (2020) 11:435. 10.1038/s41419-020-2645-3 32513988 PMC7280314

[75] Wang Y Ju C Hu J Huang K Yang L. PRMT4 overexpression aggravates cardiac remodeling following myocardial infarction by promoting cardiomyocyte apoptosis. Biochem Biophys Res Commun. (2019) 520:645–50. 10.1016/j.bbrc.2019.10.085 31627895

[76] Ito T Yadav N Lee J Furumatsu T Yamashita S Yoshida K. Arginine methyltransferase CARM1/PRMT4 regulates endochondral ossification. BMC Dev Biol. (2009) 9:47. 10.1186/1471-213X-9-47 19725955 PMC2754437

[77] Chang B Chen Y Zhao Y Bruick RK. JMJD6 is a histone arginine demethylase. Science. (2007) 318:444–7. 10.1126/science.1145801 17947579

[78] Mimura I Nangaku M Kanki Y Tsutsumi S Inoue T Kohro T. Dynamic change of chromatin conformation in response to hypoxia enhances the expression of GLUT3 (SLC2A3) by cooperative interaction of hypoxia-inducible factor 1 and KDM3 *Mol Cell Biol*. (2012) 32:3018–32. 10.1128/MCB.06643-11 22645302 PMC3434521

[79] Chakraborty D Cui W Rosario GX Scott RL Dhakal P Renaud SJ. HIF-KDM3A-MMP12 regulatory circuit ensures trophoblast plasticity and placental adaptations to hypoxia. Proc Natl Acad Sci USA. (2016) 113:E7212–21. 10.1073/pnas.1612626113 27807143 PMC5135375

[80] Guenantin AC Jebeniani I Leschik J Watrin E Bonne G Vignier N. Targeting the histone demethylase LSD1 prevents cardiomyopathy in a mouse model of laminopathy. J Clin Investig. (2021) 131:e136488. 10.1172/JCI136488 33393499 PMC7773358

[81] Papait R Serio S Pagiatakis C Rusconi F Carullo P Mazzola M. Histone methyltransferase G9a is required for cardiomyocyte homeostasis and hypertrophy. Circulation. (2017) 136:1233–46. 10.1161/CIRCULATIONAHA.117.028561 28778944

[82] Harachi M Masui K Honda H Muragaki Y Kawamata T Cavenee WK. Dual regulation of histone methylation by mTOR complexes controls glioblastoma tumor cell growth via EZH2 and SAM. Mol Cancer Res. (2020) 18:1142–52. 10.1158/1541-7786.MCR-20-0024 32366675

[83] Ishi Y Takamiya S Seki T Yamazaki K Hida K Hatanaka KC. Prognostic role of H3K27M mutation, histone H3K27 methylation status, and EZH2 expression in diffuse spinal cord gliomas. Brain Tumor Pathol. (2020) 37:81–8. 10.1007/s10014-020-00369-9 32529280

[84] Xu YH Liu K Yan J Wang HP Wu HY. [Function and mechanism of histone demethytransferase Jmjd3 mediated regulation of Th1/Th2 balance through epigenetic modification in pre-eclampsia]. Zhonghua Bing Li Xue Za Zhi. (2020) 49:1041–5. 10.3760/cma.j.cn112151-20200110-00024 32992420

[85] Tschirner A Palus S Hetzer R Meyer R Anker SD Springer J. Six1 is down-regulated in end-stage human dilated cardiomyopathy independently of Ezh2. ESC Heart Fail. (2014) 1:154–9. 10.1002/ehf2.12017 27668088 PMC5024036

[86] Neele AE Chen HJ Gijbels MJJ van der Velden S Hoeksema MA Boshuizen MCS. Myeloid Ezh2 deficiency limits atherosclerosis development. Front Immunol. (2020) 11:594603. 10.3389/fimmu.2020.594603 33574814 PMC7871783

[87] Meng XD Yao HH Wang LM Yu M Shi S Yuan ZX. Knockdown of GAS5 inhibits atherosclerosis progression via reducing EZH2-mediated ABCA1 transcription in ApoE^(−/−)^ mice. Mol Ther Nucleic Acids. (2020) 19:84–96. 10.1016/j.omtn.2019.10.034 31830648 PMC6926212

[88] Davis FM Tsoi LC Melvin WJ den Dekker A Wasikowski R Joshi AD. Inhibition of macrophage histone demethylase JMJD3 protects against abdominal aortic aneurysms. J Exp Med. (2021) 218:e20201839. 10.1084/jem.20201839 33779682 PMC8008365

[89] Bernt KM Zhu N Sinha AU Vempati S Faber J Krivtsov AV. MLL-rearranged leukemia is dependent on aberrant H3K79 methylation by DOT1L. Cancer Cell. (2011) 20:66–78. 10.1016/j.ccr.2011.06.010 21741597 PMC3329803

[90] Meister S Hahn L Beyer S Kuhn C Jegen M von Schonfeldt V. Epigenetic modification via H3K4me3 and H3K9ac in human placenta is reduced in preeclampsia. J Reprod Immunol. (2021) 145:103287. 10.1016/j.jri.2021.103287 33662848

[91] Stewart MD Lopez S Nagandla H Soibam B Benham A Nguyen J. Mouse myofibers lacking the SMYD1 methyltransferase are susceptible to atrophy, internalization of nuclei and myofibrillar disarray. Dis Models Mech. (2016) 9:347–59. 10.1242/dmm.022491 26935107 PMC4833328

[92] Berkholz J Eberle R Boller K Munz B. siRNA-mediated inhibition of skNAC and Smyd1 expression disrupts myofibril organization: Immunofluorescence and electron microscopy study in C2C12 cells. Micron. (2018) 108:6–10. 10.1016/j.micron.2018.02.009 29499397

[93] Stein AB Goonewardena SN Jones TA Prusick PJ Bazzi AA Belyavskaya JM. The PTIP-associated histone methyltransferase complex prevents stress-induced maladaptive cardiac remodeling. PLoS ONE. (2015) 10:e0127839. 10.1371/journal.pone.0127839 26001054 PMC4441468

[94] Yu S Li Y Zhao H Wang Q Chen P. The histone demethylase JMJD1C regulates CAMKK2-AMPK signaling to participate in cardiac hypertrophy. Front Physiol. (2020) 11:539. 10.3389/fphys.2020.00539 32625104 PMC7314990

[95] Zhang S Lu Y Jiang C. Inhibition of histone demethylase JMJD1C attenuates cardiac hypertrophy and fibrosis induced by angiotensin II. J Recept Signal Transduct Res. (2020) 40:339–47. 10.1080/10799893.2020.1734819 32122211

[96] Costantino S Paneni F Virdis A Hussain S Mohammed SA Capretti G. Interplay among H3K9-editing enzymes SUV39H1, JMJD2C and SRC-1 drives p66Shc transcription and vascular oxidative stress in obesity. Eur Heart J. (2019) 40:383–91. 10.1093/eurheartj/ehx615 29077881

[97] Thienpont B Aronsen JM Robinson EL Okkenhaug H Loche E Ferrini A. The H3K9 dimethyltransferases EHMT1/2 protect against pathological cardiac hypertrophy. J Clin Invest. (2017) 127:335–48. 10.1172/JCI88353 27893464 PMC5199699

[98] Kent OA Saha M Coyaud E Burston HE Law N Dadson K. Haploinsufficiency of RREB1 causes a Noonan-like RASopathy via epigenetic reprogramming of RAS-MAPK pathway genes. Nat Commun. (2020) 11:4673. 10.1038/s41467-020-18483-9 32938917 PMC7495420

[99] Kraft M Cirstea IC Voss AK Thomas T Goehring I Sheikh BN. Disruption of the histone acetyltransferase MYST4 leads to a Noonan syndrome-like phenotype and hyperactivated MAPK signaling in humans and mice. J Clin Invest. (2011) 121:3479–91. 10.1172/JCI43428 21804188 PMC3163944

[100] Koga H Kaji Y Nishii K Shirai M Tomotsune D Osugi T. Overexpression of Polycomb-group gene rae28 in cardiomyocytes does not complement abnormal cardiac morphogenesis in mice lacking rae28 but causes dilated cardiomyopathy. Lab Invest. (2002) 82:375–85. 10.1038/labinvest.3780432 11950896

[101] Stein EM Garcia-Manero G Rizzieri DA Tibes R Berdeja JG Savona MR. The DOT1L inhibitor pinometostat reduces H3K79 methylation and has modest clinical activity in adult acute leukemia. Blood. (2018) 131:2661–9. 10.1182/blood-2017-12-818948 29724899 PMC6265654

[102] Nguyen AT Xiao B Neppl RL Kallin EM Li J Chen T. DOT1L regulates dystrophin expression and is critical for cardiac function. Genes Dev. (2011) 25:263–74. 10.1101/gad.2018511 21289070 PMC3034901

[103] Fujii T Tsunesumi S Yamaguchi K Watanabe S Furukawa Y. Smyd3 is required for the development of cardiac and skeletal muscle in zebrafish. PLoS ONE. (2011) 6:e23491. 10.1371/journal.pone.0023491 21887258 PMC3160858

[104] Miao F Wu X Zhang L Yuan YC Riggs AD Natarajan R. Genome-wide analysis of histone lysine methylation variations caused by diabetic conditions in human monocytes. J Biol Chem. (2007) 282:13854–63. 10.1074/jbc.M609446200 17339327

[105] Paneni F Costantino S Battista R Castello L Capretti G Chiandotto S. Adverse epigenetic signatures by histone methyltransferase Set7 contribute to vascular dysfunction in patients with type 2 diabetes mellitus. Circul Cardiovasc Genet. (2015) 8:150–8. 10.1161/CIRCGENETICS.114.000671 25472959

[106] Wang F Ngo J Li Y Liu H Chen CH Saifudeen Z. Targeted disruption of the histone lysine 79 methyltransferase Dot1L in nephron progenitors causes congenital renal dysplasia. Epigenetics. (2020) 1–16. 10.1080/15592294.2020.1861168. [Epub ahead of print]. PMC8813085 33315499

[107] Bhat SS Schmidt KR Ladd S Kim KC Schwartz CE Simensen RJ. Disruption of DMD and deletion of ACSL4 causing developmental delay, hypotonia, and multiple congenital anomalies. Cytogenet Genome Res. (2006) 112:170–5. 10.1159/000087531 16276108

[108] Wang J Li P Xu X Zhang B Zhang J. MicroRNA-200a inhibits inflammation and atherosclerotic lesion formation by disrupting EZH2-mediated methylation of STAT3. Front Immunol. (2020) 11:907. 10.3389/fimmu.2020.00907 32655542 PMC7324475

[109] Das M Das S Lekli I Das DK. Caveolin induces cardioprotection through epigenetic regulation. J Cell Mol Med. (2012) 16:888–95. 10.1111/j.1582-4934.2011.01372.x 21707918 PMC3822857

[110] Yang G Zhang X Weng X Liang P Dai X Zeng S. SUV39H1 mediated SIRT1 trans-repression contributes to cardiac ischemia-reperfusion injury. Basic Res Cardiol. (2017) 112:22. 10.1007/s00395-017-0608-3 28271186

[111] Lee HT Oh S Ro DH Yoo H Kwon YW. The key role of DNA methylation and histone acetylation in epigenetics of atherosclerosis. J Lipid Atheroscl. (2020) 9:419–34. 10.12997/jla.2020.9.3.419 33024734 PMC7521974

[112] Su X Wang S Zhang H Yang G Bai Y Liu P. Sulforaphane prevents angiotensin II-induced cardiomyopathy by activation of Nrf2 through epigenetic modification. J Cell Mol Med. (2021) 25:4408–19. 10.1111/jcmm.16504 33793066 PMC8093985

[113] Kumar RG Spurthi MK Kumar KG Sahu SK Rani SH. Endothelial nitric oxide synthase polymorphism G298T in association with oxidative DNA damage in coronary atherosclerosis. J Genet. (2012) 91:349–52. 10.1007/s12041-012-0183-1 23271020

[114] Kuhlencordt PJ Gyurko R Han F Scherrer-Crosbie M Aretz TH Hajjar R. Accelerated atherosclerosis, aortic aneurysm formation, and ischemic heart disease in apolipoprotein E/endothelial nitric oxide synthase double-knockout mice. Circulation. (2001) 104:448–54. 10.1161/hc2901.091399 11468208

[115] Eini F Bidadkosh A Nazarian H Piryaei A Ghaffari Novin M Joharchi K. Thymoquinone reduces intracytoplasmic oxidative stress and improves epigenetic modification in polycystic ovary syndrome mice oocytes, during *in-vitro* maturation. Mol Reprod Dev. (2019) 86:1053–66. 10.1002/mrd.23222 31209968

[116] Zhang X Tanaka K Yan J Li J Peng D Jiang Y. Regulation of estrogen receptor alpha by histone methyltransferase SMYD2-mediated protein methylation. Proc Natl Acad Sci USA. (2013) 110:17284–9. 10.1073/pnas.1307959110 24101509 PMC3808627

[117] Brown MA Sims RJIII Gottlieb PD Tucker PW. Identification and characterization of Smyd2: a split SET/MYND domain-containing histone H3 lysine 36-specific methyltransferase that interacts with the Sin3 histone deacetylase complex. Mol Cancer. (2006) 5:26. 10.1186/1476-4598-5-26 16805913 PMC1524980

[118] Voelkel T Andresen C Unger A Just S Rottbauer W Linke WA. Lysine methyltransferase Smyd2 regulates Hsp90-mediated protection of the sarcomeric titin springs and cardiac function. Biochim Biophys Acta. (2013) 1833:812–22. 10.1016/j.bbamcr.2012.09.012 23047121

[119] Cattaneo P Kunderfranco P Greco C Guffanti A Stirparo GG Rusconi F. DOT1L-mediated H3K79me2 modification critically regulates gene expression during cardiomyocyte differentiation. Cell Death Differ. (2016) 23:555–64. 10.1038/cdd.2014.199 25526092 PMC4986629

[120] Wang P Lan R Guo Z Cai S Wang J Wang Q. Histone demethylase JMJD3 mediated doxorubicin-induced cardiomyopathy by suppressing SESN2 expression. Front Cell Dev Biol. (2020) 8:548605. 10.3389/fcell.2020.548605 33117796 PMC7552667

[121] Li Y Reddy MA Miao F Shanmugam N Yee JK Hawkins D. Role of the histone H3 lysine 4 methyltransferase, SET7/9, in the regulation of NF-kappaB-dependent inflammatory genes. Relevance to diabetes and inflammation. J Biol Chem. (2008) 283:26771–81. 10.1074/jbc.M802800200 18650421 PMC2546554

[122] Chokpaisarn J Urao N Voravuthikunchai SP Koh TJ. Quercus infectoria inhibits Set7/NF-kappaB inflammatory pathway in macrophages exposed to a diabetic environment. Cytokine. (2017) 94:29–36. 10.1016/j.cyto.2017.04.005 28408068 PMC5469283

[123] Fujimaki K Ogihara T Morris DL Oda H Iida H Fujitani Y. SET7/9 enzyme regulates cytokine-induced expression of inducible nitric-oxide synthase through methylation of lysine 4 at histone 3 in the islet beta cell. J Biol Chem. (2015) 290:16607–18. 10.1074/jbc.M115.661777 25995453 PMC4505414

[124] Chen J Guo Y Zeng W Huang L Pang Q Nie L. ER stress triggers MCP-1 expression through SET7/9-induced histone methylation in the kidneys of db/db mice. Am J Physiol Renal Physiol. (2014) 306:F916–925. 10.1152/ajprenal.00697.2012 24452638

[125] Ai S Yu X Li Y Peng Y Li C Yue Y. Divergent requirements for EZH1 in heart development versus regeneration. Circ Res. (2017) 121:106–12. 10.1161/CIRCRESAHA.117.311212 28512107 PMC5527745

[126] Kook H Seo SB Jain R. EZ switch from EZH2 to EZH1: histone methylation opens a window of cardiac regeneration. Circ Res. (2017) 121:91–4. 10.1161/CIRCRESAHA.117.311351 28684617

[127] Numakuraand S Uozaki H. Low MLL2 protein expression is associated with fibrosis in early stage gastric cancer. In Vivo. (2021) 35:603–9. 10.21873/invivo.12297 33402515 PMC7880768

[128] Issaeva I Zonis Y Rozovskaia T Orlovsky K Croce CM Nakamura T. Knockdown of ALR (MLL2) reveals ALR target genes and leads to alterations in cell adhesion and growth. Mol Cell Biol. (2007) 27:1889–903. 10.1128/MCB.01506-06 17178841 PMC1820476

[129] Glaser S Schaft J Lubitz S Vintersten KF. van der Hoeven, Tufteland KR, Aasland R, et al. Multiple epigenetic maintenance factors implicated by the loss of Mll2 in mouse development. Development. (2006) 133:1423–32. 10.1242/dev.02302 16540515

[130] Glaser S Lubitz S Loveland KL Ohbo K Robb L Schwenk F. The histone 3 lysine 4 methyltransferase, Mll2, is only required briefly in development and spermatogenesis. Epigenet Chromatin. (2009) 2:5. 10.1186/1756-8935-2-5 19348672 PMC2674429

[131] Goldsworthy M Absalom NL Schroter D Matthews HC Bogani D Moir L. Mutations in Mll2, an H3K4 methyltransferase, result in insulin resistance and impaired glucose tolerance in mice. PLoS ONE. (2013) 8:e61870. 10.1371/journal.pone.0061870 23826075 PMC3691224

[132] Yang Y Hao H Wu X Guo S Liu Y Ran J. Mixed-lineage leukemia protein 2 suppresses ciliary assembly by the modulation of actin dynamics and vesicle transport. Cell Disc. (2019) 5:33. 10.1038/s41421-019-0100-3 31263570 PMC6591415

[133] Yuan W Xie J Long C Erdjument-Bromage H Ding X Zheng Y. Heterogeneous nuclear ribonucleoprotein L Is a subunit of human KMT3a/Set2 complex required for H3 Lys-36 trimethylation activity *in vivo*. J Biol Chem. (2009) 284:15701–7. 10.1074/jbc.M808431200 19332550 PMC2708867

[134] Park G Gong Z Chen J Kim JE. Characterization of the DOT1L network: implications of diverse roles for DOT1L. Protein J. (2010) 29:213–23. 10.1007/s10930-010-9242-8 20431927

[135] Nguyen AT Taranova O He J Zhang Y. DOT1L, the H3K79 methyltransferase, is required for MLL-AF9-mediated leukemogenesis. Blood. (2011) 117:6912–22. 10.1182/blood-2011-02-334359 21521783 PMC3128482

[136] Jones B Su H Bhat A Lei H Bajko J Hevi S. The histone H3K79 methyltransferase Dot1L is essential for mammalian development and heterochromatin structure. PLoS Genet. (2008) 4:e1000190. 10.1371/journal.pgen.1000190 18787701 PMC2527135

[137] Jo SY Granowicz EM Maillard I Thomas D Hess JL. Requirement for Dot1l in murine postnatal hematopoiesis and leukemogenesis by MLL translocation. Blood. (2011) 117:4759–68. 10.1182/blood-2010-12-327668 21398221 PMC3100687

[138] FitzGerald J Moureau S Drogaris P O'Connell E Abshiru N Verreault A. Regulation of the DNA damage response and gene expression by the Dot1L histone methyltransferase and the 53Bp1 tumour suppressor. PLoS ONE. (2011) 6:e14714. 10.1371/journal.pone.0014714 21383990 PMC3044716

[139] Xu B On DM Ma A Parton T Konze KD Pattenden SG. Selective inhibition of EZH2 and EZH1 enzymatic activity by a small molecule suppresses MLL-rearranged leukemia. Blood. (2015) 125:346–57. 10.1182/blood-2014-06-581082 25395428 PMC4287641

[140] Konze KD Ma A Li F Barsyte-Lovejoy D Parton T Macnevin CJ. An orally bioavailable chemical probe of the Lysine Methyltransferases EZH2 and EZH1. ACS Chem Biol. (2013) 8:1324–34. 10.1021/cb400133j 23614352 PMC3773059

[141] Mori S Iwase K Iwanami N Tanaka Y Kagechika H Hirano T. Development of novel bisubstrate-type inhibitors of histone methyltransferase SET7/9. Bioorg Med Chem. (2010) 18:8158–66. 10.1016/j.bmc.2010.10.022 21036620

[142] Zhang C Hoang N Leng F Saxena L Lee L Alejo S. LSD1 demethylase and the methyl-binding protein PHF20L1 prevent SET7 methyltransferase-dependent proteolysis of the stem-cell protein SOX2. J Biol Chem. (2018) 293:3663–74. 10.1074/jbc.RA117.000342 29358331 PMC5846134

[143] Huang S Wang Z Zhou J Huang J Zhou L Luo J. EZH2 inhibitor GSK126 suppresses antitumor immunity by driving production of myeloid-derived suppressor cells. Cancer Res. (2019) 79:2009–20. 10.1158/0008-5472.CAN-18-2395 30737232

[144] Zhang Y Zhang Q Gui L Cai Y Deng X Li C. Let-7e inhibits TNF-alpha expression by targeting the methyl transferase EZH2 in DENV2-infected THP-1 cells. J Cell Physiol. (2018) 233:8605–16. 10.1002/jcp.26576 29768655

[145] Zhu WS Tang CM Xiao Z Zhu JN Lin QX Fu YH. Targeting EZH1 and EZH2 contributes to the suppression of fibrosis-associated genes by miR-214-3p in cardiac myofibroblasts. Oncotarget. (2016) 7:78331–42. 10.18632/oncotarget.13048 27823969 PMC5346642

[146] Lui JC Garrison P Nguyen Q Ad M Keembiyehetty C Chen W. EZH1 and EZH2 promote skeletal growth by repressing inhibitors of chondrocyte proliferation and hypertrophy. Nat Commun. (2016) 7:13685. 10.1038/ncomms13685 27897169 PMC5477487

[147] Liu Y Dai C Lei Y Wu W Liu W. Inhibition of EZH2 attenuates coronary heart disease by interacting with microRNA-22 to regulate the TXNIP/nuclear factor-kappaB pathway. Exp Physiol. (2020) 105:2038–50. 10.1113/EP088881 33026112

[148] Li R Yi X Wei X Huo B Guo X Cheng C. EZH2 inhibits autophagic cell death of aortic vascular smooth muscle cells to affect aortic dissection. Cell Death Dis. (2018) 9:180. 10.1038/s41419-017-0213-2 29416002 PMC5833461

[149] Ishiguro K Kitajima H Niinuma T Maruyama R Nishiyama N Ohtani H. Dual EZH2 and G9a inhibition suppresses multiple myeloma cell proliferation by regulating the interferon signal and IRF4-MYC axis. Cell Death Disc. (2021) 7:7. 10.1038/s41420-020-00400-0 33436557 PMC7803977

[150] Fan JD Lei PJ Zheng JY Wang X Li S Liu H. The selective activation of p53 target genes regulated by SMYD2 in BIX-01294 induced autophagy-related cell death. PLoS ONE. (2015) 10:e0116782. 10.1371/journal.pone.0116782 25562686 PMC4285553

[151] Wei X Yi X Zhu XH Jiang DS. Histone methylation and vascular biology. Clin Epigenet. (2020) 12:30. 10.1186/s13148-020-00826-4 32070413 PMC7027016

[152] Liu Z Wu X Lv J Sun H Zhou F. Resveratrol induces p53 in colorectal cancer through SET7/9. Oncol Lett. (2019) 17:3783–9. 10.3892/ol.2019.10034 30881498 PMC6403518

[153] Lai YS Chen JY Tsai HJ Chen TY Hung WC. The SUV39H1 inhibitor chaetocin induces differentiation and shows synergistic cytotoxicity with other epigenetic drugs in acute myeloid leukemia cells. Blood Cancer J. (2015) 5:e313. 10.1038/bcj.2015.37 25978433 PMC4476016

[154] Lin SH Ho WT Wang YT Chuang CT Chuang LY Guh JY. Histone methyltransferase Suv39h1 attenuates high glucose-induced fibronectin and p21(WAF1) in mesangial cells. Int J Biochem Cell Biol. (2016) 78:96–105. 10.1016/j.biocel.2016.06.021 27373678

[155] Luo Y Fan C Yang M Dong M Bucala R Pei Z. CD74 knockout protects against LPS-induced myocardial contractile dysfunction through AMPK-Skp2-SUV39H1-mediated demethylation of BCLB. Br J Pharmacol. (2020) 177:1881–97. 10.1111/bph.14959 31877229 PMC7070165

[156] Schweizer S Harms C Lerch H Flynn J Hecht J Yildirim F. Inhibition of histone methyltransferases SUV39H1 and G9a leads to neuroprotection in an *in vitro* model of cerebral ischemia. J Cereb Blood Flow Metab. (2015) 35:1640–7. 10.1038/jcbfm.2015.99 25966950 PMC4640311

[157] Tamura R Doi S Nakashima A Sasaki K Maeda K Ueno T. Inhibition of the H3K4 methyltransferase SET7/9 ameliorates peritoneal fibrosis. PLoS ONE. (2018) 13:e0196844. 10.1371/journal.pone.0196844 29723250 PMC5933785

[158] Zhang QJ Tran TAT Wang M Ranek MJ Kokkonen-Simon KM Gao J. Histone lysine dimethyl-demethylase KDM3A controls pathological cardiac hypertrophy and fibrosis. Nat Commun. (2018) 9:5230. 10.1038/s41467-018-07173-2 30531796 PMC6286331

[159] Cascella B Lee SG Singh S Jez JM Mirica LM. The small molecule JIB-04 disrupts O_2_ binding in the Fe-dependent histone demethylase KDM4A/JMJD2A. Chem Commun. (2017) 53:2174–7. 10.1039/C6CC09882G 28144654 PMC5511625

[160] Kim MS Cho HI Yoon HJ Ahn YH Park EJ Jin YH. JIB-04, A small molecule histone demethylase inhibitor, selectively targets colorectal cancer stem cells by inhibiting the Wnt/beta-catenin signaling pathway. Sci Rep. (2018) 8:6611. 10.1038/s41598-018-24903-0 29700375 PMC5919936

[161] Kristensen LH Nielsen AL Helgstrand C Lees M Cloos P Kastrup JS. Studies of H3K4me3 demethylation by KDM5B/Jarid1B/PLU1 reveals strong substrate recognition *in vitro* and identifies 2,4-pyridine-dicarboxylic acid as an *in vitro* and in cell inhibitor. FEBS J. (2012) 279:1905–14. 10.1111/j.1742-4658.2012.08567.x 22420752

[162] Wang Y Li Y Guo C Lu Q Wang W Jia Z. ISL1 and JMJD3 synergistically control cardiac differentiation of embryonic stem cells. Nucleic Acids Res. (2016) 44:6741–55. 10.1093/nar/gkw301 27105846 PMC5001586

[163] Guo Z Lu J Li J Wang P Li Z Zhong Y. JMJD3 inhibition protects against isoproterenol-induced cardiac hypertrophy by suppressing beta-MHC expression. Mol Cell Endocrinol. (2018) 477:1–14. 10.1016/j.mce.2018.05.009 29753027

[164] Hu Q Chen J Zhang J Xu C Yang S Jiang H. IOX1, a JMJD2A inhibitor, suppresses the proliferation and migration of vascular smooth muscle cells induced by angiotensin II by regulating the expression of cell cycle-related proteins. Int J Mol Med. (2016) 37:189–96. 10.3892/ijmm.2015.2393 26530537

[165] Abdizadeh R Heidarian E Hadizadeh F Abdizadeh T. QSAR modeling, molecular docking and molecular dynamics simulations studies of lysine-specific demethylase 1 (LSD1) inhibitors as anticancer agents. Anti Cancer Agents Med Chem. (2020). 10.2174/1871520620666200721134010. [Epub ahead of print]. 32698753

[166] Heand R Kidder BL. H3K4 demethylase KDM5B regulates global dynamics of transcription elongation and alternative splicing in embryonic stem cells. Nucleic Acids Res. (2017) 45:6427–41. 10.1093/nar/gkx251 28402433 PMC5499819

[167] Xuand J Kidder BL. KDM5B decommissions the H3K4 methylation landscape of self-renewal genes during trophoblast stem cell differentiation. Biol Open. (2018) 7:bio031245. 10.1242/bio.031245 29748167 PMC5992522

[168] Shokri G Doudi S Fathi-Roudsari M Kouhkan F Sanati MH. Targeting histone demethylases KDM5A and KDM5B in AML cancer cells: a comparative view. Leuk Res. (2018) 68:105–11. 10.1016/j.leukres.2018.02.003 29602065

[169] Backe MB Jin C Andreone L Sankar A Agger K Helin K. The lysine demethylase KDM5B regulates islet function and glucose homeostasis. J Diabetes Res. (2019) 2019:5451038. 10.1155/2019/5451038 31467927 PMC6701283

[170] Hong F Zhao M Zhang L Feng L. Inhibition of Ezh2 *in vitro* and the decline of Ezh2 in developing midbrain promote dopaminergic neurons differentiation through modifying H3K27me3. Stem Cells Dev. (2019) 28:649–58. 10.1089/scd.2018.0258 30887911

